# N-Terminal domain homologs of the orange carotenoid protein increase quenching of cyanobacterial phycobilisomes

**DOI:** 10.1093/plphys/kiae531

**Published:** 2024-10-04

**Authors:** Damien I Sheppard, Roberto Espinoza-Corral, Sigal Lechno-Yossef, Markus Sutter, Amanda Arcidiacono, Edoardo Cignoni, Lorenzo Cupellini, Benedetta Mennucci, Cheryl A Kerfeld

**Affiliations:** MSU-DOE Plant Research Laboratory, Michigan State University, East Lansing, MI 48824, USA; Department of Biochemistry and Molecular Biology, Michigan State University, East Lansing, MI 48824, USA; MSU-DOE Plant Research Laboratory, Michigan State University, East Lansing, MI 48824, USA; Department of Biochemistry and Molecular Biology, Michigan State University, East Lansing, MI 48824, USA; MSU-DOE Plant Research Laboratory, Michigan State University, East Lansing, MI 48824, USA; Department of Biochemistry and Molecular Biology, Michigan State University, East Lansing, MI 48824, USA; MSU-DOE Plant Research Laboratory, Michigan State University, East Lansing, MI 48824, USA; Environmental Genomics and Systems Biology Division, Lawrence Berkeley National Laboratory, Berkeley, CA 94720, USA; Department of Chemistry and Industrial Chemistry, University of Pisa, Pisa 56124, Italy; Department of Chemistry and Industrial Chemistry, University of Pisa, Pisa 56124, Italy; Department of Chemistry and Industrial Chemistry, University of Pisa, Pisa 56124, Italy; Department of Chemistry and Industrial Chemistry, University of Pisa, Pisa 56124, Italy; MSU-DOE Plant Research Laboratory, Michigan State University, East Lansing, MI 48824, USA; Department of Biochemistry and Molecular Biology, Michigan State University, East Lansing, MI 48824, USA; Environmental Genomics and Systems Biology Division, Lawrence Berkeley National Laboratory, Berkeley, CA 94720, USA; Molecular Biophysics and Integrated Bioimaging Division, Lawrence Berkeley National Laboratory, Berkeley, CA 94720, USA

## Abstract

Stress exerted by excess captured light energy in cyanobacteria is prevented by the photoprotective activity of the orange carotenoid protein (OCP). Under high light, the OCP converts from an orange, inactive form (OCP^O^) into the red form (OCP^R^) that binds to and quenches the phycobilisome (PBS). Structurally, the OCP consists of 2 domains: the N-terminal effector domain and a C-terminal regulatory domain. Structural analysis of the OCP-PBS complex showed that the N-terminal domains of an OCP dimer interact with the PBS core. These N-terminal OCP domains have single-domain protein paralogs known as helical carotenoid proteins (HCPs). Using PBS quenching assays, we show that the HCP4 and HCP5 homologs efficiently quench PBS fluorescence in vitro, surpassing the quenching ability of the OCP. This is consistent with computational quantum mechanics/molecular mechanics results. Interestingly, when using a maximum quenching concentration of OCP with PBSs, HCP5 addition further increases PBS quenching. Our results provide mechanistic insight into the quenching capacity and roles of HCP4 and HCP5 in cyanobacteria, suggesting that they are more than simply functionally redundant to the OCP.

## Teaser

The HCP4 homolog of the photoprotective orange carotenoid protein (OCP) quenches phycobilisomes in cyanobacteria as efficiently as OCP, whereas the HCP5 homolog surpasses OCP quenching capacity.

## Introduction

The predominant mode of photoprotection in photosynthetic organisms is nonphotochemical quenching (NPQ). This mechanism involves dissipating the excess energy absorbed by light harvesting complexes, such as the phycobilisome (PBS) of cyanobacteria (Adir et al. 2020). In many cyanobacteria, NPQ is mediated by the orange carotenoid protein (OCP) (Kirilovsky and Kerfeld 2016; Kerfeld et al. 2017), which binds to the PBS to mediate NPQ (Domínguez-Martín et al. 2022; Sauer et al. 2024). Structurally, the OCP consists of 2 domains (Kerfeld et al. 2003): a regulatory C-terminal domain (CTD), a member of the NTF2-like superfamily pfam02136 and an all-alpha helical N-terminal domain (NTD) acting as the effector domain (pfam09150), unique to cyanobacteria (Leverenz et al. 2014). The photoactivation of OCP (conversion of the inactive orange form of the protein, OCP^O^, into the red active form, OCP^R^) is predicated on a 12 Å shift of the carotenoid molecule from a position between the N-terminal and CTDs of OCP^O^, translocating it completely into the NTD of the OCP^R^ (Leverenz et al. 2015). The recent elucidation of the structure of a PBS-OCP complex from *Synechocystis* sp. PCC 6803 (hereafter *Synechocystis*) showed that 2 OCP^R^ form a dimer via their CTDs while the NTDs interact with the bilin-binding allophycocyanin (APC) proteins of the PBS core (Domínguez-Martín et al. 2022). The binding mode is conserved in PBS with tricylindrical cores (Domínguez-Martín et al. 2022; Kerfeld and Sutter 2024), but would require some structural rearrangements to bind to the PBS in cyanobacteria with pentacylindrical cores (Domínguez-Martín et al. 2022).

Phylogenetic studies using available cyanobacterial genomic sequences revealed 3 families of the OCP (Bao et al. 2017b; Slonimskiy et al. 2022; Sluchanko et al. 2024) OCP1, OCP2, and OCPX (now renamed to OCP3). Likewise, since its structural characterization, it has been known that cyanobacterial genomes contain single gene homologs to both the NTD and CTD of the OCP (Kerfeld et al. 2003; Kerfeld 2004a, b). Homologs of the CTD are described as CTD-like carotenoid proteins (CCPs or CTDHs) (Moldenhauer et al. 2017; Muzzopappa et al. 2017; Harris et al. 2018; Slonimskiy et al. 2019) and have been shown to bind a carotenoid across a CTD dimer (Lechno-Yossef et al. 2017; Dominguez-Martin et al. 2020). NTD homologs are known as helical carotenoid proteins (HCPs) (Melnicki et al. 2016). They have also been shown to bind a carotenoid, preferentially canthaxanthin (CAN) (Yang et al. 2023), and can be classified into 9 distinct clades (Melnicki et al. 2016; Bao et al. 2017a). It has been proposed that the full-length OCP originated from a gene fusion event of CCP and HCP genes (Kerfeld et al. 2003; Kerfeld 2004a, b; Kerfeld et al. 2017; Lechno-Yossef et al. 2017). When expressing synthetically separated CTD and NTD from OCP, both synthetic proteins are able to bind carotenoid to form an OCP-like heterodimer (Lechno-Yossef et al. 2017; Moldenhauer et al. 2017).

Frequently, HCPs are found in organisms that also encode an OCP. In *Anabaena* sp. PCC 7120 (hereafter *Anabaena*), there are 4 HCPs (HCP1, HCP2, HCP3 and HCP4) in addition to OCP1 (Lopez-Igual et al. 2016). Crystal structures have confirmed the structural similarity of the HCPs to the NTD of the OCP (Melnicki et al. 2016; Dominguez-Martin et al. 2019; Sklyar et al. 2024); however, only HCP4 has been shown to quench the PBS in vitro (Lopez-Igual et al. 2016).

Here, we investigate the quenching ability of HCP4 and HCP5 with both pentacylindrical and tricylindrical core PBSs and situate our findings in a structural context. Specifically, we compare the quenching ability of HCP4 (from *Anabaena*), HCP5 (from *Spirulina subsalsa* PCC 9445) and OCP1 (from *Anabaena* and *Synechocystis*) to quench *Anabaena* and *Synechocystis* PBS (corresponding to pentacylindrical and tricylindrical PBS cores, respectively) (Domínguez-Martín et al. 2022; Jiang et al. 2023). Our results show that both HCPs are as efficient in quenching PBS fluorescence as OCP1, with HCP5 being the most efficient. Surprisingly, when using a maximum quenching concentration of photoactivated OCP1, the addition of HCP5 further increases the quenching of a tricylindrical PBS. However, this effect is not seen with HCP4. The higher PBS quenching obtained by the addition of HCP5 in the presence of OCP1 suggests that HCP5 either binds additional sites in the PBS not occupied by OCP or intrinsically quenches more effectively. Quantum mechanical calculations on a model of HCP5-PBS complex provide a structural basis for the enhanced quenching.

## Results

### The distribution of HCP4 and HCP5 among cyanobacteria and structural comparison with OCP1

**Figure 1.**
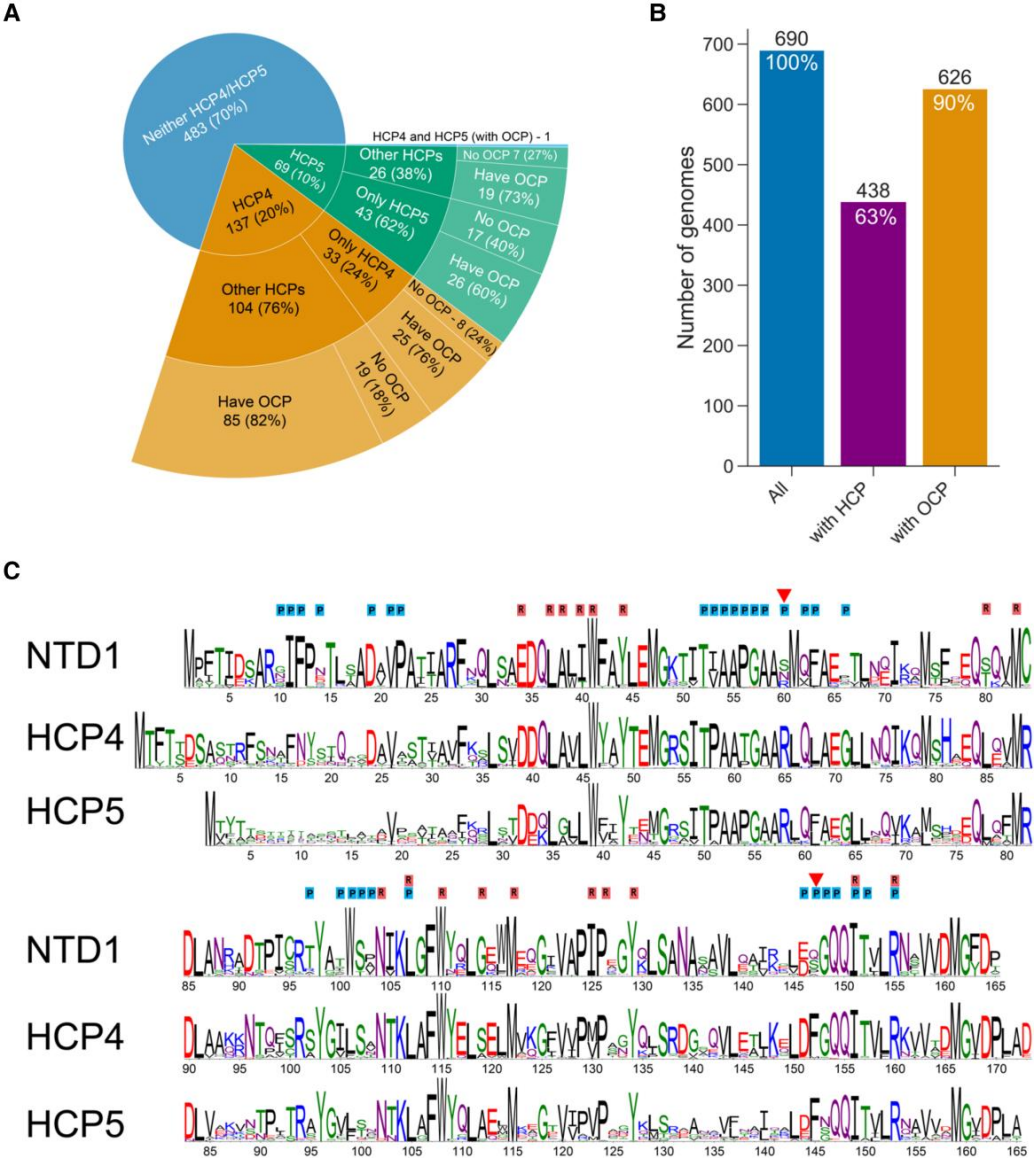
OCP-related gene distribution in cyanobacterial genomes and sequence comparison with the OCP. **A)** Distribution of HCP4, HCP5, other HCPs, and OCP in 690 cyanobacterial genomes. The number of genomes in each group is shown with the percent of its parent group. **B)** Summary of numbers of genomes analyzed that contain any HCP (middle bar) or any OCP (right bar). **C)** Comparison of sequence conservation logo of the NTD of OCP1 from 295 cyanobacterial species, 88 HCP4 sequences, and 57 sequences of HCP5. Residues of OCP^R^ that were shown to be within 4 Å of the PBS are labeled with P (blue). Residues that are within 4 Å of the carotenoid in OCP^R^ (cpcR residues) are labeled with R (red). Residues that appear to be involved in PBS interaction and are different between OCP and the HCPs are marked with a red triangle.

We selected 690 genomes from the Integrated Microbial Genomes (IMG) database (minimizing redundancy) and tallied their complement of OCP-related genes. OCP genes were found in 90% of the genomes and HCP genes in 63% of the genomes (Fig. 1B), highlighting their prevalence among cyanobacteria. In this data set, 10% (69 genomes) contain HCP5 but not HCP4 and almost twice as many (137 genomes) contain HCP4 but not HCP5 (Fig. 1A). Among all HCPs, HCP1, HCP2, HCP3, and HCP4 are the most common (Melnicki et al. 2016), and in our database, they were identified in 32%, 29%, 39%, and 20% of the genomes, respectively (Supplementary Table S1). While a large portion of genomes containing HCP4 contain additional HCP paralogs from other HCP clades (76% of the HCP4-containing genomes), the majority of analyzed genomes that contain a single or multiple copies of HCP5 genes (62% of the genomes) did not encode any other HCP paralog (Fig. 1A). Many cyanobacteria that contain HCP5 also have an OCP. Of 70 HCP5-containing genomes, 46 also encode OCP (Fig. 1A). HCP4 is even more frequently found in genomes that contain an OCP (*ocp* is present in 111 out of the 138 HCP4-containing genomes, Fig. 1A). Only a single organism in our database (*Pleurocapsa minor* PCC 7327) contains genes encoding both HCP4 and HCP5. This thermophile is a nitrogen fixing, salt-tolerant unicellular organism that also contains OCP2 and HCP7 (Fig. 1A, Supplementary Table S1).

Many of the functionally important residues of the OCP-NTD are conserved in HCP4 and HCP5 (Fig. 1C). The largest deviation between the NTD and HCP4/5 sequences is found in the N-terminal region, which in OCP is the N-terminal extension (NTE), a helical segment that contacts the CTD in the OCP^O^ form but is released upon photoactivation (Kerfeld et al. 2003; Gupta et al. 2015; Domínguez-Martín et al. 2022). Residues in this region have also been found to interact peripherally at the bottom cylinder binding site of the PBS in the quenching complex (Domínguez-Martín et al. 2022). In HCP5, there is very little overall conservation of the N-terminal 25 residues (Fig. 1C).

Many of the residues known to be important for interaction with the PBS are also conserved between *Synechocystis* OCP and HCP4/5 (Fig. 1C). Notable differences between the PBS interacting residues are found at position 60 (*Synechocystis* OCP numbering), where a Ser is replaced by Arg in both HCPs (R65 in HCP4; R58 in HCP5), and at position 147, where a Ser is replaced by a conserved Phe in the HCPs (red triangles, Fig. 1C). Judging from the homology models, the increased size of the residues in the HCPs can be accommodated in the quenching complex, and potentially influence the binding properties favorably by increasing the number of interprotein contacts.

**Figure 2.**
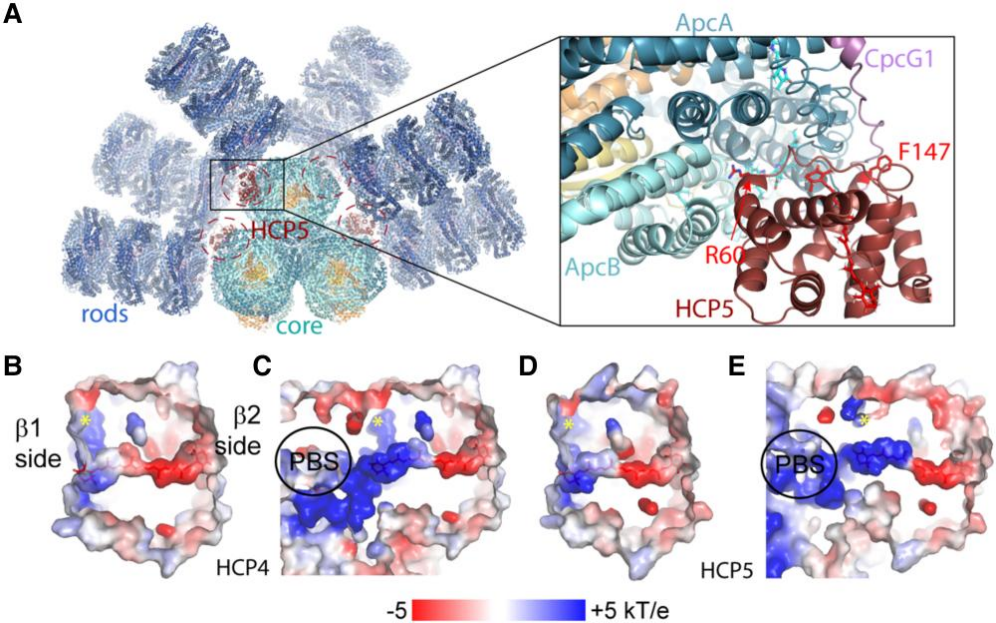
Model of the HCP5-PBS complex and electrostatic environments of the carotenoid in HCP4 and HCP5. **A)** Left: overview of an HCP5-PBS complex model with the potential 4 binding sites (highlighted with a dashed ellipse), 2 each for the bottom and top core cylinders. Right: close-up of the HCP5-PBS interaction site on the top cylinder with highlighted residues R60 and F147. Electrostatics of the carotenoid environment for homology models of HCP4 **B)**, HCP4-PBS **C)**, HCP5 **D)**, and HCP5-PBS **E)**. The position of a conserved phenylalanine in HCP4 and HCP5 (residues 152 and 145, respectively) is indicated with a yellow asterisk.

Residues within 4 Å of the carotenoid in OCP^R^ (cpcR residues; Leverenz et al. 2015) are mostly conserved in HCP4 and HCP5 (Fig. 1C). These are important in establishing the transition dipole moment (TDM) of the carotenoid to poise it for quenching (Sauer et al. 2024). Consistent with this, the electrostatic environment of the carotenoid in homology models of HCP4 and HCP5 is very similar to the carotenoid environment in *Synechocystis* OCP^R^ (Fig. 2, B to E).

### *An*HCP4 and *An*OCP1 quench *An*PBS comparably

**Figure 3.**
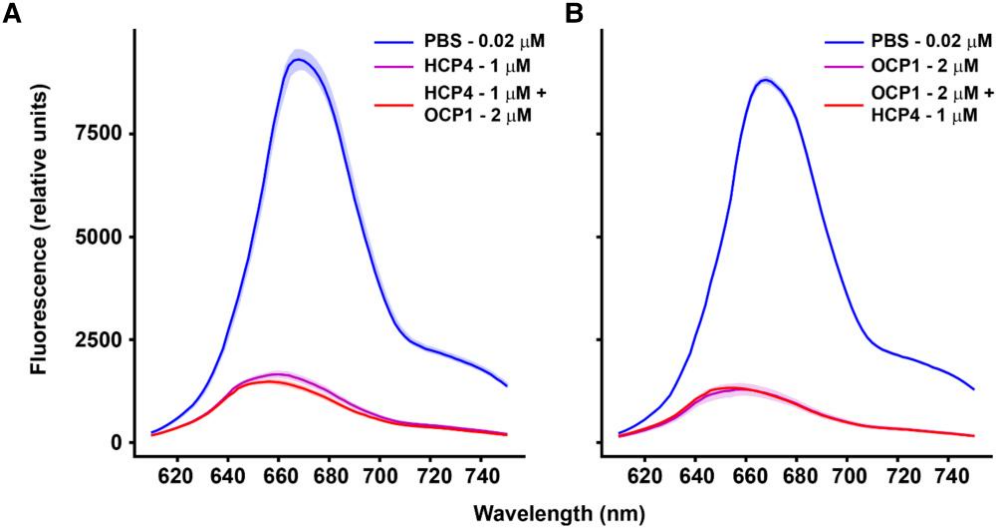
Comparison of quenching capacity between HCP4 and OCP1. *Anabaena* PBS were isolated to perform in vitro quenching experiments with either *An*OCP1 (photoactivated) and HCP4 (purified holoprotein in both cases). **A)**  *Anabaena* PBS fluorescence quenching by HCP4 followed by addition of photoactivated *An*OCP1. **B)** Photoactivated *An*OCP1 was used to quench PBS with a subsequent addition of HCP4. Values correspond to mean, and the shaded areas correspond to the Sd of 3 technical replicates in each case.

In *Anabaena*, the transcription of both OCP and HCP4 is upregulated after exposure to high light (A.M. Muro-Pastor and M. Brenes-Álvarez, unpublished). Additionally, among the 4 HCPs in *Anabaena*, only HCP4 was shown to quench PBS fluorescence in vitro (Lopez-Igual et al. 2016). We have tested the in vitro quenching capacity of the holo-HCP4 in isolation or in combination with the *Anabaena* OCP1 (herein *An*OCP1) with *Anabaena* PBS (herein *An*PBS). Maximum fluorescence quenching was achieved by 1 *µ*m HCP4 or 2 *µ*m  *An*OCP1 (Supplementary Fig. S1). No effect on quenching was observed when they were added sequentially at their respective maximum quenching concentrations (Fig. 3). These results suggest that the HCP4 and *An*OCP1 compete for the same binding sites on their native/cognate pentacylindrical PBS and quench in a similar manner.

### HCP5 is a more efficient quencher of tricylindrical PBS than OCP1

**Figure 4.**
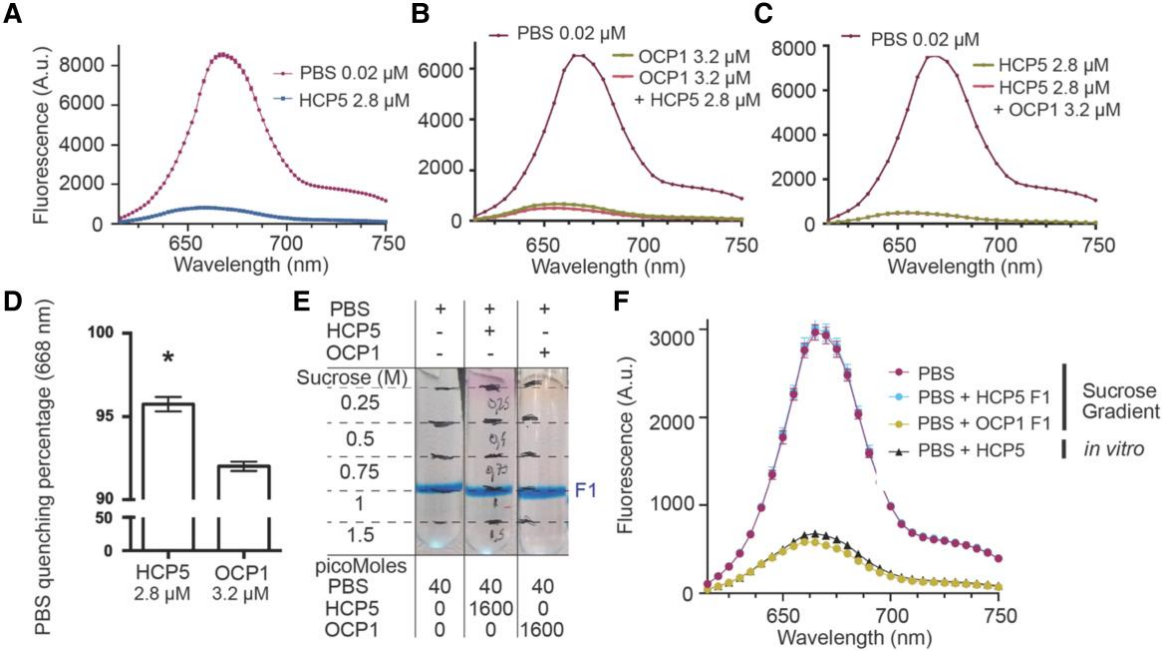
Comparison of quenching capacity between HCP5 and OCP1. *Syn*PBS were isolated to perform in vitro quenching experiments with either OCP1 (photoactivated) and HCP5 (purified holoenzyme in both cases). **A)** Quenching capacity of HCP5 with *Syn*PBS; values correspond to mean and error to standard error of the mean (SEM) of 3 technical replicates. **B)** Photoactivated OCP1 was used to quench PBS with a subsequent addition (+) of HCP5; values correspond to mean and error to SEM of 2 technical replicates. **C)** PBS quenching using HCP5 with subsequent addition of photoactivated OCP1; values correspond to mean and error to SEM of 2 technical replicates. **A** to **C)** correspond to a representative experiment out of 3 biological replicates. **D)** Maximum PBS quenching percentage comparison of HCP5 and OCP1. PBS maximum fluorescence at 668 nm was used to calculate quenching percentages. Values correspond to 3 biological replicates, error bars to SEM, and an asterisk to statistical difference according to Student's *t* test (2 sided, *P* < 0.005). **E)** PBS binding assay using either photoactivated OCP1 or HCP5. Symbols + and − correspond to addition or absence, respectively. **F)** Samples from **E)** were collected (F1) to measure their fluorescence by bringing all samples to the same PBS concentration. Additionally, in vitro quenching of PBS using HCP5 is shown to demonstrate the ability of HCP5 to quench PBS. Values (arbitrary units, A.u.) correspond to 2 technical replicates and error to SEM. A representative experiment is shown out of 3 biological replicates.

We tested if recombinant holo-HCP5 from *S. subsalsa*, an organism with a tricylindrical core PBS, could quench the *Synechocystis* PBS, which is also tricylindrical (Fig. 4A). We observed quenching with a maximum capacity at a concentration of 2.8 *μ*m for HCP5 (Supplementary Fig. S2A), while a similar concentration of 2 *μ*m for S*ynechocystis* OCP1 (hereafter *Syn*OCP1), the native quencher for this PBS, was needed to obtain maximum quenching (Supplementary Fig. S2B). Interestingly, when comparing the PBS quenching capacity of HCP5 and *Syn*OCP1 using the same concentration of quencher, HCP5 shows higher quenching than *Syn*OCP1 (Supplementary Fig. S2, A and B). Similarly, when we used holo-HCP4, the concentration needed to achieve maximum quenching was 3 *μ*m (Supplementary Fig. S2C) and that of *An*OCP1 was at least 4 *μ*m (Supplementary Fig. S2D). Comparing results presented in Supplementary Figs. S1 and S2, it appears that the HCP4 and *An*OCP1 are more efficient in quenching *An*PBS fluorescence compared to *Syn*PBS.

To determine if *Syn*OCP1^R^ and HCP5 bind to the same site on the tricylindrical PBS, we assayed them in combinations. Surprisingly, when *Syn*OCP1^R^ was used to quench *Syn*PBS with subsequent addition of HCP5, quenching was further enhanced (Fig. 4B). However, in the converse experiment in which HCP5 quenching was followed with the addition of *Syn*OCP1^R^, no additional quenching was observed (Fig. 4C). Comparison of the maximum PBS quenching percentage shows that indeed HCP5 displays higher quenching capacity compared to *Syn*OCP1^R^ (Fig. 4D). This suggests that HCP5 either binds to the PBS at sites additional to those occupied by the OCP or is a stronger quencher, or both. Such enhancement effects were not observed for the *An*OCP1/HCP4-*An*PBS complex (Fig. 3). Interestingly, addition of HCP4 to *Syn*PBS quenched by either *Syn*OCP1 or *An*OCP1 did enhance the fluorescence quenching, as observed for the HCP5 (Supplementary Fig. S3), suggesting that HCP binding to the tricylindrical PBS of *Synechocystis*, an organism that does not contain HCPs, is somehow different than their binding to the pentacylindrical PBS of *Anabaena*.

To evaluate the strength of association of HCP5 with the PBS, we compared sucrose gradient profiles of the quenched (HCP5-*Syn*PBS or *Syn*OCP^R^-*Syn*PBS) complexes (Fig. 4E). When the fluorescence of the OCP^R^-*Syn*PBS complex was measured postcentrifugation, PBS fluorescence was quenched compared to *Syn*PBS without OCP^R^ in agreement with previously published results (Gwizdala et al. 2011). In contrast, the putative HCP5-*Syn*PBS complex showed no difference in fluorescence compared to *Syn*PBS alone (Fig. 4F), indicating that the HCP5 dissociated during centrifugation. This suggests that the HCP5 binds less tightly than OCP1 to the PBS.

### Computational simulation supports the stronger PBS quenching by HCP5

The higher quenching capacity of HCP5 with respect to OCP1 and in comparison to an HCP (HCP2) that is not an active quencher (Lopez-Igual et al. 2016) as a control was further investigated with calculations of the TDM for the 2 low-lying excited states of the CAN carotenoid, as well as the excitation energy transfer (EET) coupling between CAN and the bilin pigments in ApcA_1_ and ApcA_2_. The quenching rate, indeed, can be directly connected to the coupling which in turn is connected to the TDM.

The computational protocol applied here was the same used for the *Synechocystis* OCP1-PBS model (Sauer et al. 2024), which allows for a direct comparison between the 2 systems. The HCP5-PBS model (Fig. 2A) was used in a restrained molecular dynamics (restMD) simulation, in which the protein backbone was subject to a restraining potential. Due to the large dimensions of the PBS core, parts of the model were excluded from the simulation, keeping only the closest chains to HCP5 or HCP2. Different configurations of the system were used to apply a quantum mechanics/molecular mechanics (QM/MM) approach where CAN is the QM subsystem whereas the MM subsystem contains the proteins and the solvent (see Materials and methods).

**Figure 5.**
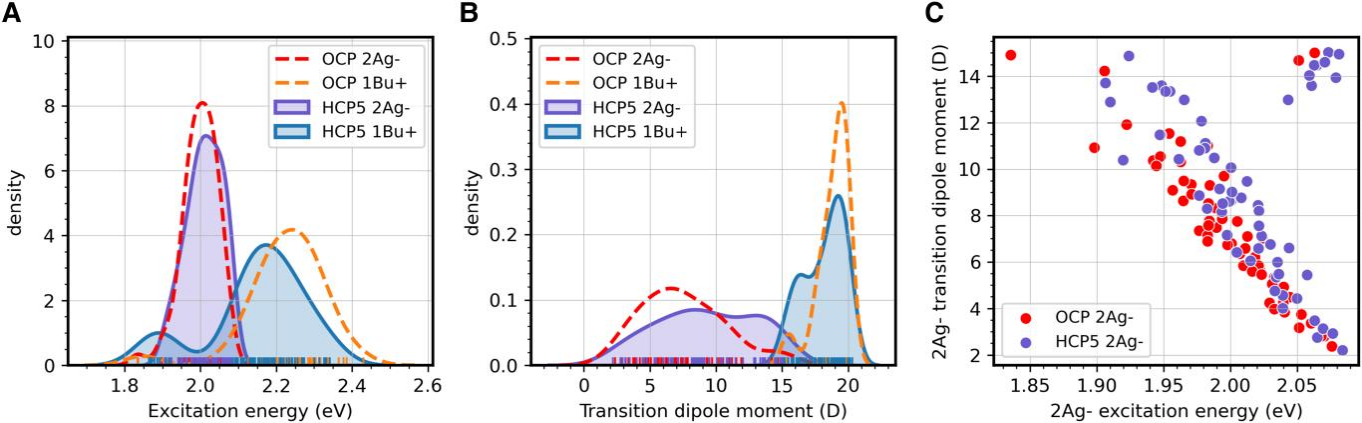
CAN excited state properties in HCP5 and OCP1. **A** and **B)** Comparison between the distribution of the excitation energy **A)** and the TDM **B)** of the dark (2A_g_^−^) and bright (1B_u_^+^) excited states of CAN in OCP1 (dashed lines) and HCP5 (solid lines). **C)** Correlation between excitation energy and TDM of the dark 2A_g_^−^ state of CAN in HCP5 (purple points) and OCP1 (red points).

We recall that in isolated CAN, the S_0_ to S_1_ transition is optically forbidden, so S_1_ is a dark state (symmetry 2A_g_^−^), while the S_2_ (1B_u_^+^) state can absorb light and therefore is bright. However, the protein environment can increase the TDM of S_1_ of CAN by mixing 2A_g_^−^ with 1B_u_^+^, substantially influencing its quenching capacity. In Fig. 5, we report the QM/MM excitation energies and the corresponding TDMs for the dark and bright states calculated along the restMD trajectories of HCP5 and OCP1. Given the restraints on the backbone, the distribution of the investigated properties only arises from the dynamics of water and external side chains, which modulate the protein electric field acting on the embedded carotenoid.

As can be seen, the energies of the dark (2A_g_^−^) and bright (1B_u_^+^) states are closer in HCP5 (the average difference between the 2 states is 0.14 eV in HCP5 and 0.23 eV in OCP1), indicating a larger mixing between the 2 states (Fig. 5A). Indeed, we observe a larger TDM for the dark state (10.0 D in HCP5 against 8.2 D in OCP1) and a corresponding lower TDM for the bright state (18.2 D in HCP5 against 18.9 D in OCP1, Fig. 5B). From a direct comparison between the excitation energy and TDM of the dark state, we can observe that in HCP5, there are more configurations in which the state is lower in energy and has a larger TDM (Fig. 5C). Notably, there are also more structures where the energy order of the dark and bright state is inverted (points on the top right corner of Fig. 5C), meaning that the mixing between the states is very high.

**Figure 6.**
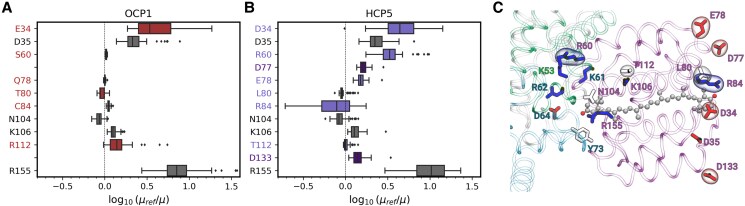
Impact of the protein environment on the 2A_g_^−^ TDM of CAN. **A** and **B)** Effect of residues on the 2A_g_^−^ TDM in OCP1 **A)** and HCP5 **B)**. Center line, median; box limits, upper and lower quartiles; whiskers, 1.5× interquartile range; points, outliers. Sample size is *N* = 60. The dashed lines represent *μ* = *μ*_ref_, i.e. zero effect. The colored boxes are referred to residues that are different in the 2 proteins. The gray boxes refer to the common residues. The missing entries for OCP1 correspond to nonpolar residues, which are not shown. **C)** Visual representation of the residues in the protein pocket of CAN in HCP5. The residues are colored by residue type: red for negatively charged residues (Asp and Glu), blue for positively charged residues (Lys and Arg), and white for polar/nonpolar residues. Label color refers to the chain: purple for HCP5, turquoise for ApcA, and green for ApcB. The residues encircled in the surface are the ones that change from OCP1 to HCP5.

The increased TDM of the dark state of CAN in HCP5, when compared to OCP1, is the result of the asymmetric charge distribution within the embedding pocket (Fig. 6). In HCP5, additional charged residues are located at the 2 ends of the CAN chain, producing a stronger electric field on the embedded CAN (Fig. 6C). Indeed, in HCP5, the positively charged R replaces S60 (*Synechocystis* NTD numbering) on the protein side adjacent to the PBS, increasing the CAN TDM (Fig. 6, A and B). On the other side of the HCP5 protein, the negatively charged D34, D77, E78, and D133 replace E34, L77, Q78, and A133, respectively (Fig. 6C). The presence of the positively charged R84 on the solvent-exposed side of HCP5 is not sufficient to counterbalance the effect of the additional negative charges on the same side (Fig. 6B). Overall, these substitutions result in an increased imbalance in the electrostatic potential in the carotenoid environment in HCP5 relative to OCP1 (Supplementary Fig. S4).

In contrast, in HCP2 which is not able to quench the PBS, the 2 TDMs are more separated than in HCP5 and OCP, indicative of a smaller mix between the 2 states (Supplementary Fig. S5). Moreover, the distribution of the TDMs of the dark state (2A_g_^−^) is centered at smaller values with respect to the other 2 proteins. Finally, the excitation energy of the bright state (1B_u_^+^) is slightly blue shifted with respect to OCP, while it appeared red shifted in HCP5, and the excitation energy of 2A_g_^−^ has a narrower distribution (Supplementary Fig. S5). The main reason for this different behavior can be found in the electrostatic configuration around CAN in the 3 different proteins. From a principal component analysis on the electrostatic potential generated by the protein pocket onto CAN's atoms, we can notice that the HCP2 potential is projected on a whole different region of OCP1 and HCP5. In Supplementary Fig. S6, we show that the relationship between the potential imbalance (ΔP) and the 2A_g_^−^ TDM is linear in all proteins. HCP2 samples values of ΔP that are closer to 0 with respect the other 2 proteins, while in HCP5, we observe the opposite trend. This could explain the reason why the TDM of 2A_g_^−^ in HCP2 does not increase as much as in the other 2 proteins.

From this analysis, we can affirm that HCP2 shows a lower degree of mixing between CAN states compared to HCP5 and OCP1 with a consequent reduction in the TDM of the dark state. This assumes that the HCP2 can bind to the PBS at the known binding site. Inspection of the HCP2 homology model at the OCP^R^ binding site shows that due to a number of amino acid substitutions, the binding of HCP2 at this site this seems unlikely, providing another explanation for why HCP2 cannot quench the PBS.

**Figure 7.**
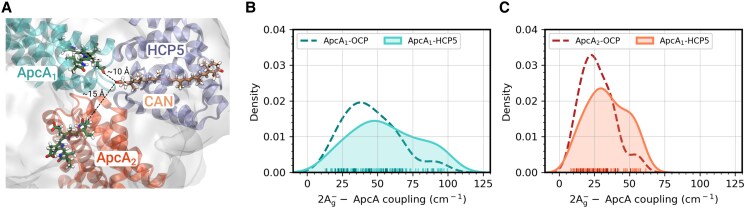
CAN-ApcA electronic couplings in HCP5 and OCP1. **A)** Visual representation of the HCP5-PBS model (CAN). **B and C)** Distribution of the CAN-PCB electronic coupling in ApcA_1_  **B)** and ApcA_2_  **C)** for both OCP1-PBS (dashed line) and HCP5-PBS (solid line).

The same QM/MM calculations along the restMD trajectories were used to compute EET couplings between the lowest excitation of CAN and the closest bilins that are located in 2 different Apc subunits, ApcA_1_ and ApcA_2_, respectively (Fig. 7A). Figure 7, B and C, shows the distribution of the couplings with both pigments, which clearly peak at larger coupling values (Fig. 7, B and C). The higher proportion of HCP5 structures with large couplings reflects the increased TDM of the dark state. The average coupling increases by about 25% for ApcA_1_ (56 vs 45 cm^−1^) and ApcA_2_ (34 vs 27 cm^−1^), implying a faster EET in HCP5-PBS and thus a more efficient quenching.

## Discussion

Quenching of the PBS is a fundamental process for preventing adverse effects of light stress in cyanobacteria. The N-terminal effector domain of the OCP that physically interacts with the PBS core (Domínguez-Martín et al. 2022) is a domain found only in cyanobacteria (Kerfeld et al. 2017). It is also found as single-domain carotenoid binding proteins known as HCPs (Melnicki et al. 2016). Phylogenetically, of the 9 HCP clades, HCP4 and HCP5 are the ones most closely related to the NTD of the OCP (Melnicki et al. 2016), consistent with their observed PBS quenching capacity (Figs. 3 and 4).

The observation of the absence of an OCP in many genomes that contain HCP5 suggests that HCP5 (Fig. 1) might be involved in NPQ in the absence of OCP. However, no NPQ was observed under bright blue light in vivo in *Thermosynechococcus elongatus* BP-1, an organism that contains an HCP most closely related to HCP5 but lacks OCP (Boulay et al. 2008; Lopez-Igual et al. 2016), suggesting that HCP5 quenching if extant in BP-1 must be elicited by another mechanism. Notably, the transcript level of HCP5 in this strain was shown to be upregulated under medium light, which is the theoretical saturating irradiance (1,190 *µ*mol m^−2^ s^−1^) but not under high light (1,995 *µ*mol m^−2^ s^−1^) when compared with 197 *µ*mol m^−2^ s^−1^ (Bernstein et al. 2017). Functional analyses of the HCP homologs from *Anabaena* showed the ability of HCP4 to quench pentacylindrical (*Anabaena*) and tricylindrical (*Synechocystis*) PBS (Lopez-Igual et al. 2016). Sequence comparison among HCP4, HCP5, and NTD showed high conservation with the NTD regions known to physically interact with PBS (Fig. 1C) with HCP5 being most closely related. The HCP5 of *Spirulina* has 52% identity with the *Syn*NTD1 and 50% identity with *An*NTD1, while the HCP4 of *Anabaena* has 50% and 54% identity with *Syn*NTD1 and *An*NTD1, respectively. In contrast, *An*HCP2 shows only 38% and 34% sequence identity with AnNTD1 and *An*HCP4, respectively. These results add to the enigma surrounding the function of the HCPs; the paralogs typically show distinct regulatory patterns, hinting at distinct functions other than quenching (Giner-Lamia et al. 2014; Melnicki et al. 2016; Ho et al. 2017; Yang et al. 2019; Llewellyn et al. 2020; Petrescu et al. 2021; Yang et al. 2023).

Previous reports have provided evidence of *An*HCP4 quenching PBS (Lopez-Igual et al. 2016); however, the quenching capacity among HCP homologs with OCP1 has not been analyzed yet. We compare the quenching efficiency among *An*HCP4, HCP5, and OCP1 (from *Anabaena* and *Synechocystis*), and PBS quenching experiments show that *An*HCP4 quenches to the same capacity as *An*OCP1 (Fig. 3). Using maximum quenching concentrations for both *An*HCP4 and *An*OCP1, the maximum molar quenching capacity of *An*HCP4 (1 *µ*m) requires double the *An*OCP1 concentration (2 *µ*m) (Supplementary Fig. S1). Strikingly, after reaching maximum quenching capacity of *Syn*PBS by *Syn*OCP1, the addition of HCP5 further increased *Syn*PBS quenching (Fig. 4).

In silico comparison of the electrostatic environment in the vicinity of the carotenoid for HCPs and OCP supports their functional differences in PBS quenching (Fig. 2, B to E). The CAN binding pocket is characterized by several charged residues that do not have a counterpart in OCP1; these increase the net electric field acting on CAN (Fig. 6). The HCP5 electrostatics induces the mixing of the dark and bright excited states, increasing the TDM of the former. As we have found recently (Sauer et al. 2024), the CAN in OCP-PBS has a larger TDM arising from the mixing of the dark and bright states, which results in substantial EET couplings that explain the efficiency of OCP as a quencher. The larger EET couplings found for HCP5 then suggest that it is a more effective quencher than OCP1. Indeed, QM/MM calculations of HCP5 and OCP1 show that EET couplings between CAN and Apc bilins are higher for HCP5, suggesting that it presents an intrinsically higher quenching capacity (Fig. 7). By comparison, in HCP2, the TDM is significantly lower than in either HCP5 or OCP1. This and differences in primary structure that may preclude binding to the known quencher binding site may explain the inability of HCP2 to quench (Lopez-Igual et al. 2016). In contrast, due to the lack of a CTD, HCP5 could potentially bind to additional sites in the PBS core where the OCP^R^ dimer is unable to bind due to steric hindrance (Domínguez-Martín et al. 2022).

On the other hand, the stability of the OCP1-PBS and HCP5-PBS complexes differs. Stability of the complexes through sucrose gradient separation indicated that the OCP1 interaction with PBS is more stable than of HCP5 (Fig. 4). The higher affinity of the *Syn*OCP1 to *Syn*PBS is likely due to increased avidity because of having 2 binding sites resulting from the CTD dimerization. In contrast, due to the lack of the CTDs, all 4 PBS binding sites only have a single attachment point to the HCPs (Fig. 2A). This may indicate that under conditions in which the HCPs are involved in photoprotection, their activity is primarily controlled by protein binding. Despite the observation that the HCP5 has a higher PBS quenching capacity compared to OCP1, the stability of the OCP^R^ dimer with PBS could provide a longer PBS quenching over time in the cell. Future structural insights on the interaction of HCP5 with PBS could reveal the structural basis of its differences with OCP1.

## Materials and methods

### Bioinformatic survey of cyanobacterial genomes for the presence of HCP5 and sequence comparison between HCP5 and OCP1

A database of 690 cyanobacterial genomes was retrieved from the IMG database (https://img.jgi.doe.gov/). A total of 1,972 protein sequences containing either pfam09150, characteristic to the NTD of the OCP, and 9,900 sequences containing pfam02136, characteristic to the CTD of the OCP, were retrieved (April 2022). Query HMMs for each of the 9 HCP clades described by Melnicki et al. (2016) or 4 OCP clades (Bao et al. 2017b) were generated using the hmmbuild function of the HMMER suite (Potter et al. 2018). The sequence database was scored with hmmsearch and resulted in 1,155 and 752 sequences identified as HCP and OCP, respectively. The complement of OCP-related genes in each of the analyzed cyanobacterial genomes is presented in Supplementary Table S1. The data in Supplementary Table S1 were used to generate Fig. 1A.

A total of 57 HCP5 sequences longer than 120 amino acids, 88 sequences identified as HCP4, and 295 OCP1 sequences were aligned separately using MAFFT (LINS-i method) (Katoh et al. 2005). The alignments were curated manually, and the OCP alignment was trimmed to include only the NTD of the OCP and exclude the flexible linker and the CTD. Sequence conservation logos were generated using WebLogo (Crooks et al. 2004).

### Molecular cloning, protein expression, and purification

Clones used in this study are described in Supplementary Table S2.

*Syn*OCP1 of *Synechocystis* sp. PCC 6803 with a C-terminal His-tag was expressed in CAN-producing *Escherichia coli* and purified as described in Domínguez-Martín et al. (2022). *An*OCP1 of *Anabaena* sp. PCC 7120 with a C-terminal His-tag was generated by subcloning *all3149* into pET28a, generating pMB10 (Supplementary Table S2). The plasmid was expressed in BL21 (DE3) containing pAC-CANthipi (to allow production of CAN), and the protein was purified by affinity chromatography followed by hydrophobic interaction chromatography as for the *Syn*OCP1.

HCP5 of *S. subsalsa* PCC 9445 (Spi9445_2287) cloned with a C-terminal Strep-tag (pDS61) and pCDF-all4941-Ctag, a clone of HCP4 of *Anabaena* sp. PCC 7120 with a C-terminal His-tag (Lopez-Igual et al. 2016), were expressed in BL21 (DE3) along with pAC-CANthipi. Protein expression and affinity purification were performed as described in Lechno-Yossef et al. (2017). To separate the holo- from the apoprotein of HCP5, the elution fractions from StrepTrap column were diluted 2-fold with buffer A (50 mm Tris, pH 8) to decrease the salt concentration down to 100 mm, and loaded on a MonoQ 5/50 GL column (GE Healthcare, Chicago, IL, USA) equilibrated with buffer A. The column was then washed with 10 column volumes (CVs) of buffer A and 5 CVs of 10% buffer B (50 mm Tris, pH 8.0; 1 m NaCl). A slow gradient was started and manually stopped when peaks started to elute. The holo-HCP5 peak was eluted at 10.5% to 11% of buffer B, just before the apo-HCP5 peak was eluted at the same salt concentration (Supplementary Fig. S7, A and B). A similar approach was employed for separation of holo-HCP4, but a complete buffer exchange into buffer A using Amicon 10 kDa concentrator was carried out before loading on the MonoQ. For HCP4, the holoprotein was eluted in the flow through and column wash (Supplementary Fig. S7, C and D). Protein concentration was determined based on the maximum absorbance of CAN at 530 nm with CAN extinction coefficient of 118,000 m^−1^ cm^−1^ and a 1:1 ratio of HCP5:CAN or HCP4:CAN.

### PBS purification from *Synechocystis* sp. PCC 6803 and *Anabaena* sp. PCC 7120 and binding assays

*Synechocystis* and *Anabaena* PBS were isolated as described in Espinoza-Corral et al. (2024). Briefly, wild-type strains of *Synechocystis* and *Anabaena* were grown under normal conditions until stationary phase before cells were harvested and resuspended in phosphate buffer (0.8 m, pH 7.5) supplemented with protease inhibitor cocktail (Sigma). Cells were broken by 4 French pressing rounds followed by the addition of Triton X-100 (1% v/v) and incubated for 15 min under darkness and room temperature. The lysate was separated by centrifugation (30,000 × *g*, 22 °C for 30 min) to rescue the soluble fraction containing PBS that was subsequently centrifuged again (42,000 rpm, 22 °C for 1 h) recovering the soluble PBS in the supernatant using a syringe. This supernatant was loaded onto a discontinuous sucrose gradient composed of 1.5, 1, 0.75, 0.5, and 0.25 m phases in phosphate buffer (0.8 m, pH 7.5) and separated by centrifugation at 25,000 rpm and room temperature overnight. PBS were recovered from the 0.75 to 1 m sucrose phases using a syringe and quantified as described by Gwizdala et al. (2011).

When performing PBS binding assays, isolated PBS from a previous sucrose gradient were diluted 5 times with phosphate buffer to reduce the sucrose concentration. Purified holo-HCP5 or photoactivated OCP1 were added (in 40 times molar ratio relative to PBS) to the diluted PBS and incubated in darkness for 1 h with rotation. The samples were then loaded onto discontinuous sucrose gradient using the same composition and method described above. Thus, PBS were rescued from the lower sucrose phases while the excess of either HCP5 or OCP1 stayed at the top of the sucrose gradient. PBS were quantified as described by Gwizdala et al. (2011).

### Fluorescence quenching assays

In vitro fluorescence measurements were conducted as described by Gwizdala et al. (2011). Purified PBS were diluted to 0.02 *µ*m in 0.8 m phosphate buffer, pH 7.5, and the emission spectrum was collected using SpectraMax M2 fluorimeter (Molecular Devices) with excitation at 580 nm. After collecting the control fluorescence spectra of unmodified PBS, various concentrations of quenchers were added at concentrations and order specified in the figure legends (Fig. 2, Supplementary Fig. S1), and the fluorescence spectrum measurement was repeated. Photoactivation of the OCP was conducted using a blue LED light (*λ*_max_ = 470 nm, Philips Lumileds LXML-PB01-0030) at 1,000 *µ*mol photons m^−2^ s^−1^.

### Structural modeling

SWISS-MODEL (swissmodel.expasy.org) was used to generate models of *Anabaena* HCP4 and *S. subsalsa* PCC 9445 HCP5 sequences based on the NTD of the *Synechocystis* OCP^R^-PBS structure (PBD ID 7SC9). This was done to more accurately obtain a PBS-bound conformation as without a template, the loop region (residues 54 to 60 in HCP5) will not be properly modeled and produce clashes with the PBS residues. The coordinates of the PBS were used from the high-resolution structure of the T cylinder binding site (PDB ID 8TPJ). For the HCP5-PBS complex, 2 residues were adjusted manually: the side chain of R155 was modified to be in the same conformation as the OCP in the high-resolution OCP-PBS complex and the side chain of R84 was adjusted to remove a clash with the carotenoid. Structures were visualized with PyMOL 2.5.2 (www.pymol.org) and electrostatic surfaces calculated with the APBS plugin of PyMOL.

### MD

MD simulations and system preparation were performed using Amber 18 (Lee et al. 2018). The model system, consisting in HCP5 and the 7 closest apoprotein chains of PBS core, was solvated in a truncated octahedron box of ∼14 nm diameter. The charge of the system was neutralized adding Na^+^ ions. In all simulation steps, the protein was described with the AMBER ff14SB force field (Maier et al. 2015) and phycocyanobilins (PCBs) with GAFF (Wang et al. 2004). Instead, CAN was described with our previously developed force field (Bondanza et al. 2020). For water, we used the TIP3P model. The entire system was minimized subject to 4 kcal mol^−1^ Å^−1^ restraints on all nonsolvent nonhydrogen atoms. Then, the system was heated gradually to 300 K in 20 ps, with the same restraints in the NVT ensemble. Finally, the box was equilibrated through a 1 ns NPT simulation using the Monte Carlo barostat implemented in Amber, with the same restraints. All simulations were run with the Langevin thermostat, a time step of 2 fs, and the SHAKE algorithm. PME electrostatics was used with a short-range cutoff of 1 nm. Two independent restMD production replicas were run for 40 ns subject to 4 kcal mol^−1^ Å^−1^ restraints on the backbone of the proteins.

### QM/MM calculations

From the 2 restMD replicas, we extracted 60 frames to be used in QM/MM calculations. The CAN molecule was replaced with a QM/MM optimized geometry within the protein environment, performed with density functional theory at the B3LYP/6-31G(d) level. The calculations of the excited states of CAN are performed with a semiempirical CI method that relies on parameters previously optimized for carotenoids (Accomasso et al. 2022). The environment is included with an electrostatic embedding QM/MM scheme, which comprehends the atoms of the protein, solvent, ions, and other cofactors.

The couplings between the pigments were calculated with the transition charges (TrEsp) method (Madjet et al. 2006). TrEsp charges are obtained by fitting the transition electrostatic potential (as obtained in QM/MM calculations) onto the pigment's atoms. The couplings between 2 chromophores A and B are computed as follows:


$$V_{\mathrm{AB}}=\overset{N_{A}}{\underset{i\in A}{\sum }}\;\overset{N_{B}}{\underset{\;j\in B}{\sum }}\frac{q_{i}^{\mathrm{tr}}q_{j}^{\mathrm{tr}}}{r_{ij}}$$

where $q_{i}^{\mathrm{tr}}$and $q_{j}^{\mathrm{tr}}$ are the TrEsp charges of atom *i* of chromophore A and atom *j* of chromophore B, and $r_{ij}$ is the distance between them. The couplings were computed in the 60 frames extracted from the restMD of HCP5-PBS. The TrEsp charges of CAN are computed for every extracted snapshot, in order to capture the fluctuations of the electric field due to the environment. The TrEsp charges of the PCBs were the same as obtained in our previous work (Sauer et al. 2024).

The effect of protein residues on CAN's TDM was determined by a systematic “turn-off of the relevant residues. The selected residues are the charged/polar residues within 10 Å of CAN in OCP1 and HCP5. The “turn-off” of the residues consists in setting to 0 the charges of their side chains and repeating the QM/MM calculations to obtain the TDM. The comparison with the reference is computed as *l*og_10_ (*µ/µ*_ref_) where *µ*_ref_ is the TDM of CAN in the “all-on” configuration of the residues. The procedure is repeated for each of the 60 frames extracted from the MD trajectories.

### Accession numbers

Sequence data from this article can be found in the GenBank/EMBL data libraries under accession numbers in Supplementary Table S1.

## Supplementary Material

kiae531_Supplementary_Data

## Data Availability

The data underlying this article are available in the article and in its online supplementary material.

## Supplementary data

The following materials are available in the online version of this article.

**Supplementary Figure S1.** Analysis of maximum quenching concentrations for HCP4 and *An*OCP1.

**Supplementary Figure S2.** Analysis of maximum quenching concentrations for HCP5, HCP4, and OCP1 from different organisms.

**Supplementary Figure S3.** Comparison of *Syn*PBS quenching capacity between HCP4 and OCP1.

**Supplementary Figure S4.** Electrostatic potential imbalance in OCP1 and HCP5.

**Supplementary Figure S5.** Distributions of TDM and excitation energy of 1B_u_^+^ and 2A_g_^−^.

**Supplementary Figure S6.** Electrostatic potential imbalance in OCP1, HCP5, and HCP2.

**Supplementary Figure S7.** HCP4 and HCP5 holo- and apoprotein purification.

**Supplementary Table S1.** Cooccurrence of OCP and HCP among cyanobacteria genomes.

**Supplementary Table S2.** Plasmid list.
