# Supplementary material for: N-Terminal domain homologs of the orange carotenoid protein increase quenching of cyanobacterial phycobilisomes
Source: Plant Physiol. 2024 Oct 4;198(1):kiae531. doi: 10.1093/plphys/kiae531 (PMC12059629; doi:10.1093/plphys/kiae531)
Supplement: kiae531_Supplementary_Data [file kiae531_supplementary_data.zip › Supplementary Figure S1S7 Updated.pdf]

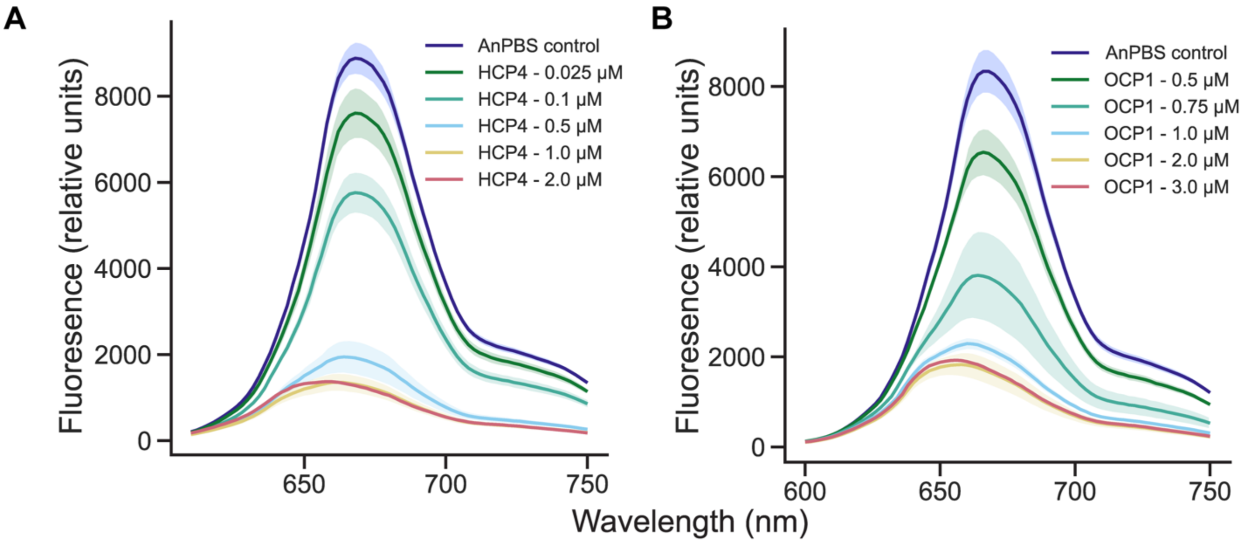

Supplementary Figure S1. Analysis of maximum quenching concentrations for HCP4 and *An*OCP1. *Anabaena* PBS were used to determine the required concentration to reach maximum quenching for HCP4 **(A)** and OCP1 **(B)**. Values correspond to mean, and the shaded areas correspond to standard deviation of three technical replicates in each case.

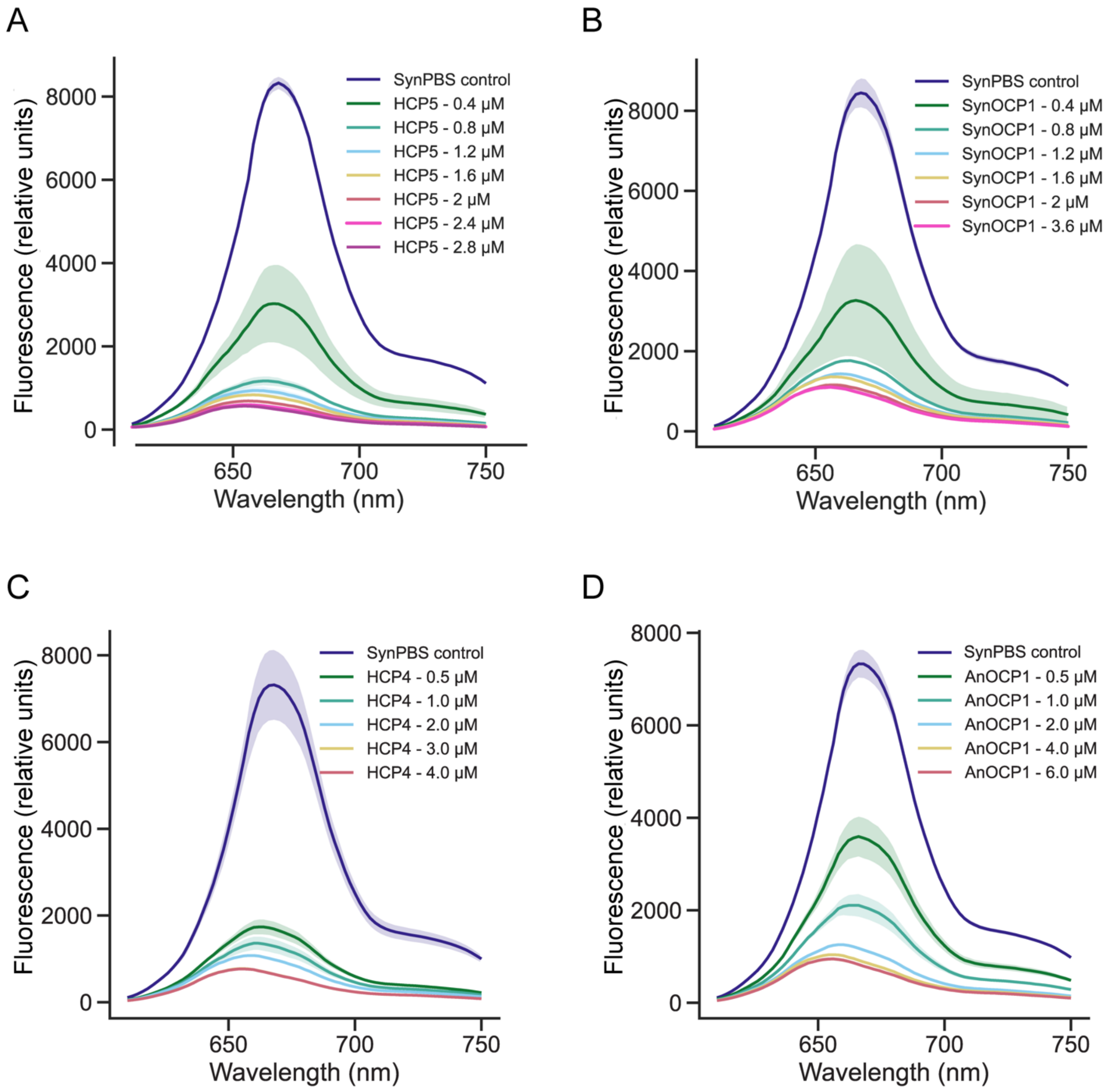

Supplementary Figure S2. Analysis of maximum quenching concentrations for HCP5, HCP4 and OCP1 from different organisms. *Synechocystis* PBS were used to determine the required concentration to reach maximum quenching for HCP5 **(A)**, SynOCP1 **(B),** HCP4 **(C)** and AnOCP1 **(D)**. PBS concentration used – 0.02 μM. Values correspond to mean, and the shaded areas correspond to standard deviation of three technical replicates in each case. A representative experiment out of three biological replicates is shown.

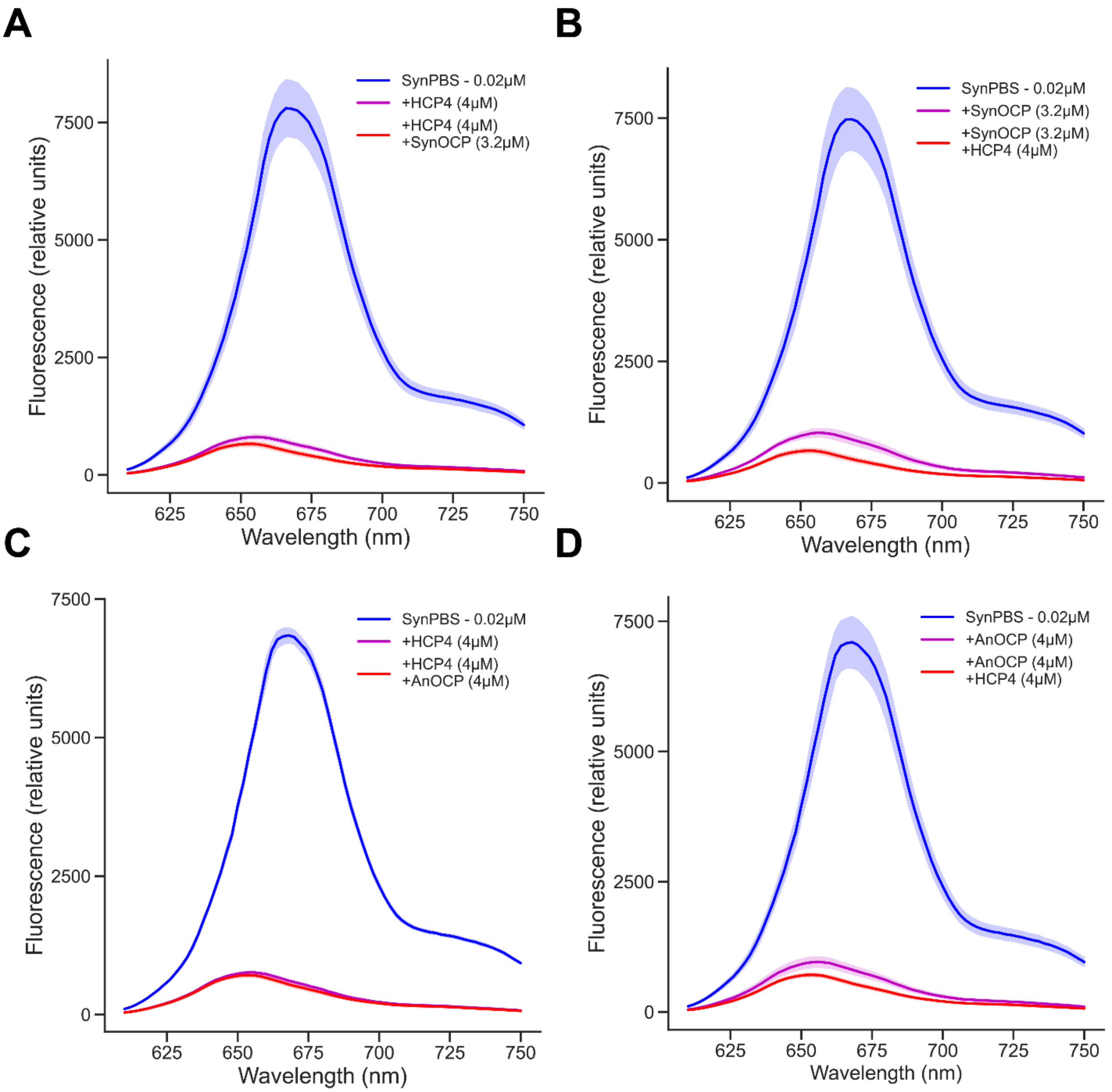

Supplementary Figure S3. Comparison of SynPBS quenching capacity between HCP4 and OCP1. *Synechocystis* PBS were isolated to perform *in-vitro* quenching experiments with either *Syn*OCP1, *AnOCP*1 and HCP4 (purified holo protein in all cases). **(A)** *Syn*PBS fluorescence quenching by HCP4 followed by addition of photoactivated *Syn*OCP1. **(B)** Photoactivated *Syn*OCP1 was used to quench *Syn*PBS with a subsequent addition of HCP4. **(C)** *Syn*PBS fluorescence quenching by HCP4 followed by addition of photoactivated *An*OCP1. **(D)** Photoactivated *An*OCP1 was used to quench *Syn*PBS with a subsequent addition of HCP4. Values correspond to mean, and the shaded areas correspond to standard deviation of three technical replicates in each case.

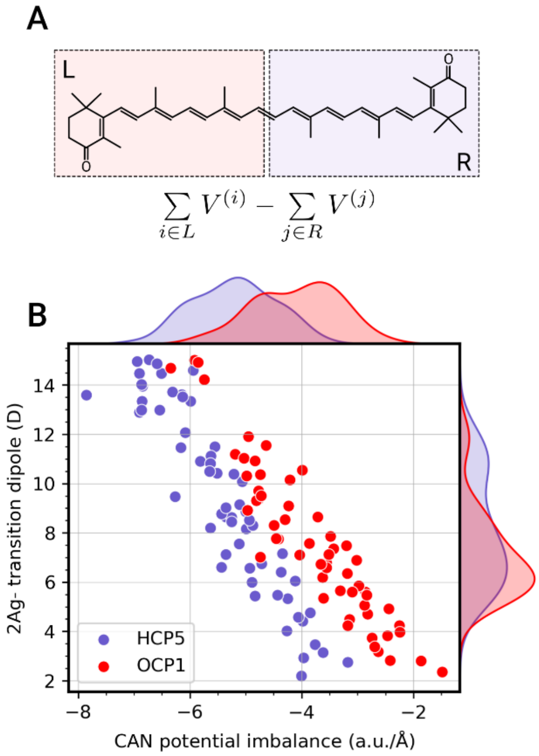

Supplementary figure S4. Electrostatic potential imbalance in OCP1 and HCP5. (A) Visual representation and mathematical formula for the calculation of the electrostatic potential imbalance. The electrostatic potential is generated by the charges of the environment at the position of the atoms of canthaxanthin (CAN). The potential imbalance is calculated as the difference between the cumulative potential on the left side (L) and the right side (R) of CAN. **(B)** Correlation between the imbalance of environment electrostatic potential and $2A_g^-$ TDM in the binding pocket of CAN in HCP5 (purple points) and OCP1 (red points). The distributions of transition dipole moment (TDM) and potential imbalance are also reported.

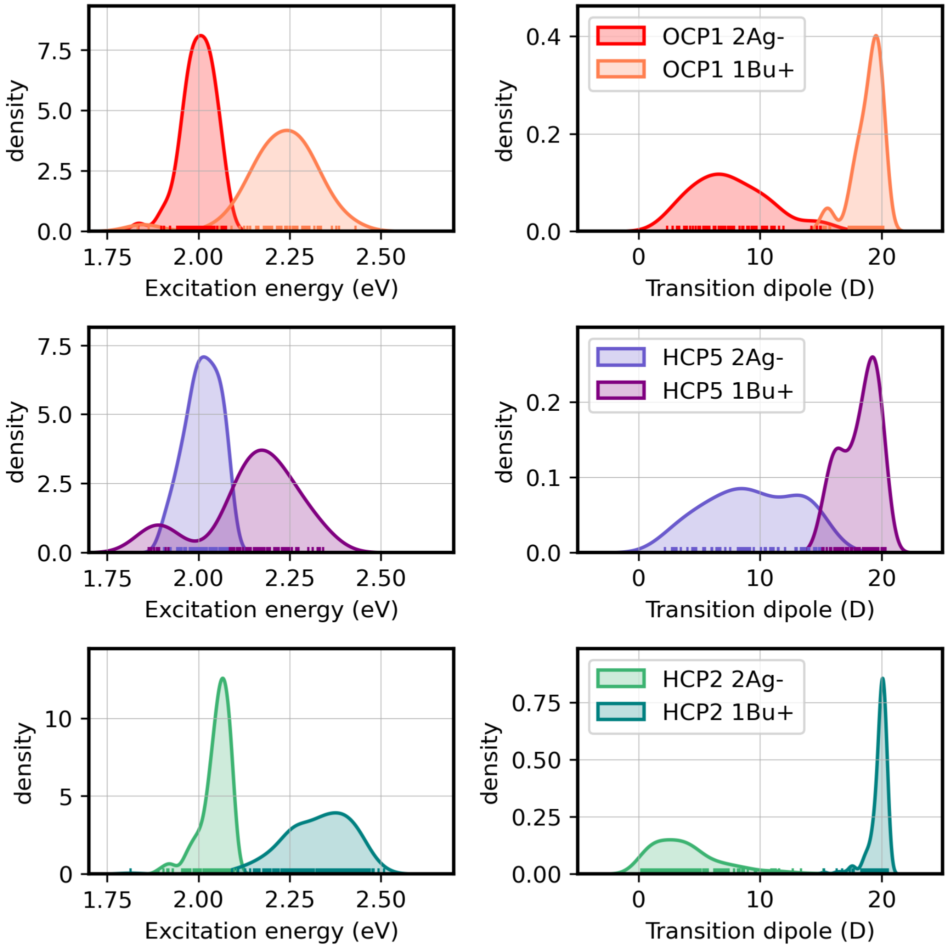

Supplementary figure S5. Distributions of TDM and excitation energy of 1Bu+ and 2Ag-. From top to bottom, we compare excitation properties in OCP, HCP5 and HCP2. In the left panels we show the distributions of the excitation energies of the first two states of CAN, on the right the transition dipole moment (TDM).

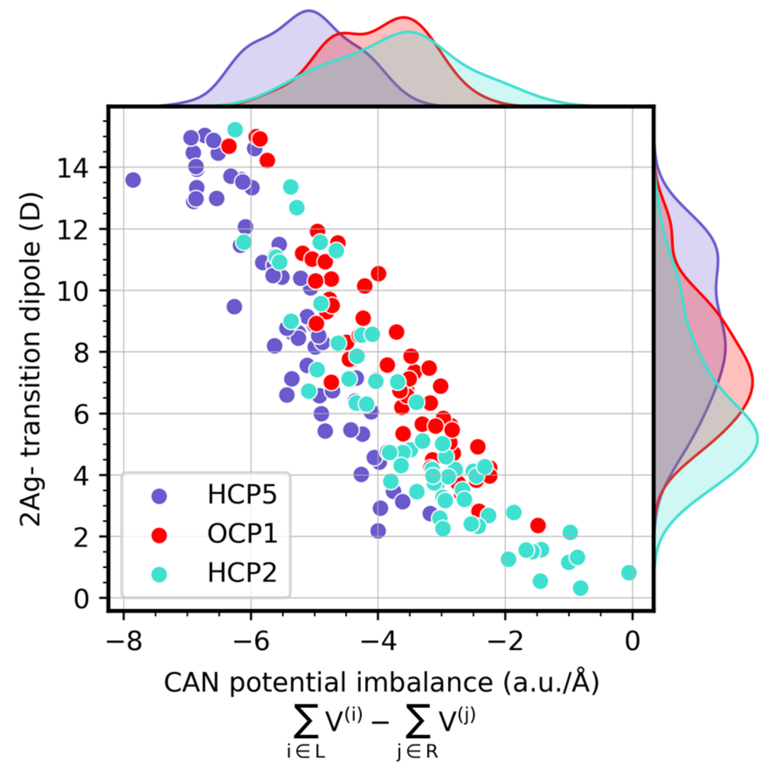

Supplementary figure S6. Electrostatic potential imbalance in OCP1, HCP5 and HCP2. Correlation between the imbalance of environment electrostatic potential and $2A_g^-$ TDM (transition dipole moment) in the binding pocket of canthaxanthin (CAN) in HCP5 (purple points), OCP1 (red points) and HCP2 (green points). The distributions of TDM and potential imbalance are also reported.

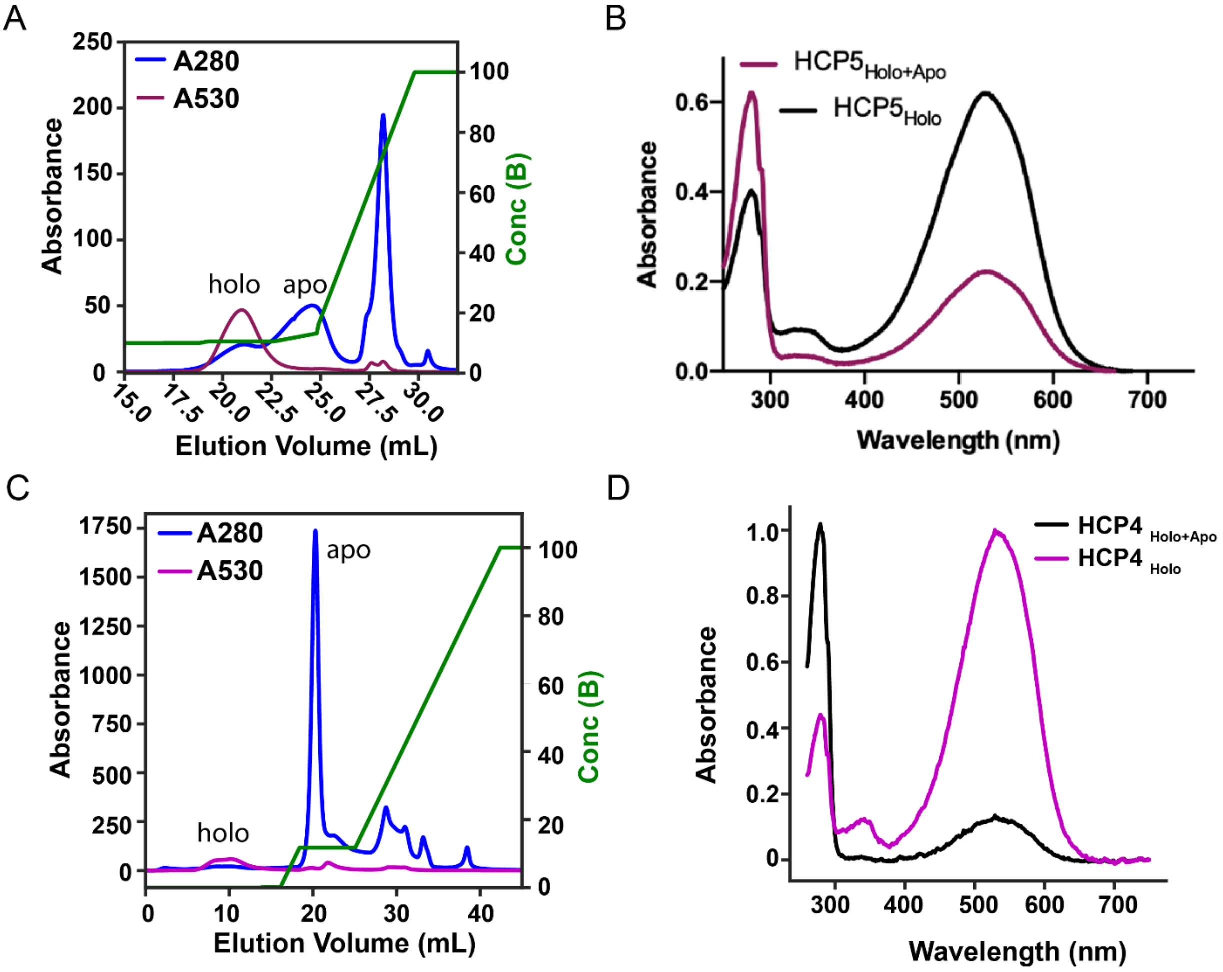

Supplementary Figure S7. HCP4 and HCP5 holo and apo protein purification. (A) Chromatograms of MonoQ separation between Apo- and Holo-HCP5. Absorbance at 280 nm (blue) and 530 nm (purple) are shown. **(B)** Absorbance spectra comparison of HCP5 holo protein after MonoQ purification and a mixture of HCP4 holo and apo proteins after elution from Strep affinity column. **(C)** Chromatograms of MonoQ separation between Apo- and Holo-HCP4. Absorbance at 280 nm (blue) and 530 nm (magenta) are shown. **(D)** Absorbance spectra comparison of HCP4 holo protein after MonoQ purification and a mixture of HCP4 holo and apo proteins after elution from Ni affinity column. Representative purification example for each protein is shown.
