# Supplementary material for: N-Terminal domain homologs of the orange carotenoid protein increase quenching of cyanobacterial phycobilisomes
Source: Plant Physiol. 2024 Oct 4;198(1):kiae531. doi: 10.1093/plphys/kiae531 (PMC12059629; doi:10.1093/plphys/kiae531)
Supplement: kiae531_Supplementary_Data [file pp.01066.2024-s06.pdf › pp.01066.2024-s06.pdf]

Plant Physiology®

N-terminal domain homologs of the orange carotenoid protein increase quenching of cyanobacterial phycobilisomes

Damien Sheppard, Roberto Espinoza-Corral, Sigal Lechno-Yossef, Markus Sutter, Amanda Arcidiacono, Edoardo Cignoni, Lorenzo Cupellini, Benedetta Mennucci, and Cheryl Kerfeld

Review Timeline:

Submission Date:	10-May-2024
Editorial Decision:	10-Jun-2024
Resubmission Received:	17-Jul-2024
Editorial Decision:	30-Aug-2024
Revision Received:	16-Sep-2024
Accepted:	17-Sep-2024

08-Jun-2024

Prof. Cheryl A Kerfeld
MSU/LBNL
Biochemistry
East Lansing, Michigan 48824

RE: N-terminal domain homologs of the orange carotenoid protein increase quenching of cyanobacterial phycobilisomes

Dear Dr. Cheryl Kerfeld:

Thank you for giving us the opportunity to review your manuscript for publication in the Plant Physiology. The reviewers have found the manuscript of interest, but have raised a number of concerns that will require your attention. As these concerns are of some gravity and will entail substantial changes to the manuscript, you are likely to need additional time to address them. I must therefore reject the manuscript in its present form. Nonetheless, if you are able to address the points raised in review, I would welcome your resubmission of a manuscript that incorporates your responses.

In particular, I direct your attention to the following comments for consideration in a resubmitted manuscript.

Reviewer #2 is requesting absorption spectra of the HCP samples studied, and additional computational analysis of a non-quenching HCP homolog to strengthen the finding. She/He also recommends to specify the novelty of this study by crediting a previous paper showing quenching ability of HCP. Reviewer #1 is pointing to inconsistency in text description of Fig. 1A. Please also consider including the "unpublished data" as supplementary.

Please note that any resubmission will be treated as a new entry to the system and is likely to go out for review.

If you do have any queries or concerns, you are welcome to contact me in the first instance.

Thank you again for allowing Plant Physiology to review your work. We look forward to hearing from you soon.

Sincerely,

Editor, Plant Physiology

-- Reviewer comments:
Reviewer #1 (Comments for the Author):

The manuscript updated the knowledge about HCP4 and HCP5, can quenching PBS, individually. according the genomic screen, authors reported distribution of HCP4 and HCP5, and their association with OCP. Then the QM/MM calculation supported the energy mechanisms during PBS quenching. However, the manuscript cannot be accepted as current form.
1. page 4, lines 108-122, the text description did not match with he number presented in Fig 1A. Not sure which one is correct, e.g. in the text, HCP5 10% (70 genomes), but fig 10% (69 genomes),
HCP4, 20% (138 genomes) vis 20% 137 genomes;
Line 113, ...80% of the HCP4-containing genomes but in Fig 76% (?).
Line 114, ---HCP5 (61% of genomes..), in Fig A, 62% (?)
Also line 119, 34% ... 10%, .. seem also not align well with the figure 1A
The results statement (Line 133-135) should be highlighted in Fig 1C to make clear.
Fig 2B-E, did not reflect the Phe replacements (in HCP4 and HCP5). it would be helpful to label (or highlight) Phe replacement there.
Line 148-149, cited 'unpublished data). If it is important for your experiment design, should author consider to include them as support data here, instead of "unpublished data"
Additionally, Fig 4 is difficult to read due to small fonts.

Reviewer #2 (Comments for the Author):

This work presents incremental in vitro evidence for quenching induced by homologs of the NTD of OCP. Several significant concerns require the authors' attention:

-The capability of HCP to quench phycobilisomes is already known. This was demonstrated by Kerfeld's group in collaboration

with Rocío López-Igual et al., in Plant Physiology 2016. Although briefly mentioned in the text, the paper still suggests a major discovery is the quenching ability of HCP. For instance, the abstract states, "Using phycobilisome quenching assays, we show that the HCP4 and HCP5 homologs are able to quench PBS fluorescence in vitro as efficiently or more efficiently than the OCP." The prior demonstration that HCP4 can quench should be more clearly acknowledged in the paper, and the actual novelty of the findings needs clearer articulation.

-Given the journal's scope, the physiological relevance of the finding that HCP5 enhances the quenching efficiency of OCP in Synechocystis phycobilisomes is ambiguous, particularly as HCP5 originates from a different organism than the phycobilisomes used in vitro. This point should be clarified.

-The paper novelty seem to lie primarily on the computational investigation rather than the physiological one. The novelty of this study stems from demonstrating that HCP5, unlike other HCP homologs, can induce quenching because of its electrostatics. Therefore, it is essential to substantiate the effectiveness of the quantum mechanics methods used for HCP5. A comparative analysis employing identical methodologies on at least one HCP homolog that does not induce quenching and also on HCP4 which induces quenching, is necessary to convincingly demonstrate that these methods can accurately identify the origins of quenching.

-The absorption spectra for each sample studied are missing, omitting crucial data for understanding if the spectroscopic properties of either HCP or of the phycobilisomes are altered during complex preparation.

-The reason for the emission spectra shifting to blue upon quenching induction should be elucidated by the authors, and normalized emission spectra should be provided for clearer understanding.

-of major concern, the authors neglect relevant recent literature on OCP, while citing 20 out of 35 references from the works of the corresponding author.

Reviewer comments:
Reviewer #1 (Comments for the Author):

The manuscript updated the knowledge about HCP4 and HCP5, can quenching PBS, individually. according the genomic screen, authors reported distribution of HCP4 and HCP5, and their association with OCP. Then the QM/MM calculation supported the energy mechanisms during PBS quenching. However, the manuscript cannot be accepted as current form.
page 4, lines 108-122, the text description did not match with the number presented in Fig 1A. Not sure which one is correct, e.g. in the text, HCP5 10% (70 genomes), but fig 10% (69 genomes), HCP4, 20% (138 genomes) vis 20% 137 genomes;

We thank the reviewer for appreciating the various new contributions of this paper and the careful reading that helps us to further clarify the findings.

The difference of one genome between the text and the figure is due to counting the one genome that contains both HCP4 and HCP5 in both groups. In **Figure 1A**, this one genome is counted separately and shown on top of the HCP5 section as one sentence "HCP4 and HCP5 (with OCP) -1". We clarified this further in lines 109-110 "*In this dataset, 10% (69 genomes) contain HCP5 but not HCP4 and almost twice as many (137 genomes) contain HCP4 but not HCP5 (**Figure 1A**)"*, and lines 120-122 *"Only a single organism in our database (Pleurocapsa minor PCC 7327) contains genes encoding both HCP4 and HCP5. This thermophile is a nitrogen fixing, salt tolerant unicellular organism which also contains OCP2 and HCP7 (**Figure 1A, Table S1**)*"

Line 113, ...80% of the HCP4-containing genomes but in Fig 76% (?).
Line 114, ---HCP5 (61% of genomes..), in Fig A, 62% (?)

We thank the reviewer for noticing this inconsistency in our original manuscript. We corrected the text in lines 113-116 "*While a large portion of genomes containing HCP4 contain additional HCP paralogs from other HCP clades (76% of the HCP4-containing genomes), the majority of analyzed genomes that contain a single or multiple copies of HCP5 genes (62% of the genomes), did not encode any other HCP paralog (**Figure 1A**)*".

Also line 119, 34% ... 10%, .. seem also not align well with the figure 1A

Indeed, we corrected the text and further clarify by simplifying the description of our observations in lines 116-120 *"Many cyanobacteria that contain HCP5 also have an OCP. Of 70 HCP5 containing genomes, 46 also encode OCP (**Figure 1A**). HCP4 is even more often found in genomes that contain an OCP (ocp is present in 111 out of the 138 HCP4-containing genomes, **Figure 1A**)".*

The results statement (Line 133-135) should be highlighted in Fig 1C to make clear.

We appreciate this suggestion. Red triangles were added where a Ser is replaced by a conserved Phe in **Figure 1C**. This is now reflected in the text in lines 135-136 "*(red triangles, **Figure 1C**)".*

Fig 2B-E, did not reflect the Phe replacements (in HCP4 and HCP5). it would be helpful to label (or highlight) Phe replacement there.

We appreciate the suggestion of the reviewer. A modified **Figure 2B-E** now denotes the position of the phenylalanine residue using an asterisk.

Line 148-149, cited 'unpublished data). If it is important for your experiment design, should author consider to include them as support data here, instead of "unpublished data"

This statement, "In *Anabaena,* the transcription of both OCP and HCP4 is upregulated after exposure to high light (Alicia Muro Pastor and Manuel Brenes-Álvarez, unpublished)" is not crucial to our experimental design; it was merely provided as context. These RNA-Seq data are the basis for the PhD work of a student that is analyzing global responses to light/darkness in Anabaena, so it will take some time to publish this entire dataset. If deemed necessary, we can strike out this statement, as it does not affect our findings or experimental design.

Additionally, Fig 4 is difficult to read due to small fonts.

We modified **Figure 4** using a larger font size as suggested by the reviewer.

Reviewer #2 (Comments for the Author):

This work presents incremental in vitro evidence for quenching induced by homologs of the NTD of OCP. Several significant concerns require the authors' attention:

-The capability of HCP to quench phycobilisomes is already known. This was demonstrated by Kerfeld's group in collaboration with Rocío López-Igual et al., in Plant Physiology 2016. Although briefly mentioned in the text, the paper still suggests a major discovery is the quenching ability of HCP. For instance, the abstract states, "Using phycobilisome quenching assays, we show that the HCP4 and HCP5 homologs are able to quench PBS fluorescence in vitro as efficiently or more efficiently than the OCP." The prior demonstration that HCP4 can quench should be more clearly acknowledged in the paper, and the actual novelty of the findings needs clearer articulation.

We agree that the ability of HCP4 to quench the PBS was known. We acknowledge this important evidence in lines 284-286 "*Functional analyses of the HCP homologues from Anabaena showed the ability of HCP4 to quench pentacylindrical (Anabaena) and tricylindrical (Synechocystis) PBS (Lopez-Igual et al., 2016)*".

The novelty of our findings includes the surprising observation that HCP5 is a quencher of the 6803 PBS, and much more importantly a more efficient quencher compared to HCP4 and OCP1 at their maximum quenching concentrations. We highlight this observation in lines 296-299 "*Previous reports have shown evidence of AnHCP4 quenching PBS (Lopez-Igual et al., 2016), however the quenching capacity among HCP homologues with OCP1 has not been analyzed yet. We compare the quenching efficiency among AnHCP4, HCP5 and OCP1 (from Anabaena and Synechocystis)".* Moreover, HCP5 provides additional quenching to that of OCP1 with its cognate PBS (**Figure 4**).

Additionally, the association of the quencher with PBS showed that HCP homologues do not interact as tightly as OCP1 does, suggesting different binding mechanisms. We indicate this in lines 322-333 "*On the other hand, the stability of the OCP1-PBS and HCP5-PBS complexes differ. Stability of the complexes through sucrose gradient separation indicated that the OCP1 interaction with PBS is more stable than of HCP5 (**Figure 4**). The higher affinity of the SynOCP1 to SynPBS is likely due to increased avidity due to having two binding sites resulting from the CTD dimerization. In contrast, due to the lack of the CTDs, all four PBS binding sites only have a single attachment point to the HCPs (**Figure 2A**). This may indicate that under conditions in which the HCPs are involved in photoprotection, their activity is primarily controlled by protein binding. Despite the observation that the HCP5 has a higher PBS quenching capacity compared to OCP1, the stability of the OCP^R dimer with PBS could provide a longer PBS quenching over time in the cell. Future structural insights on the interaction of HCP5 with PBS could reveal the structural basis of its differences with OCP1*".

-Given the journal's scope, the physiological relevance of the finding that HCP5 enhances the quenching efficiency of OCP in Synechocystis phycobilisomes is ambiguous, particularly as HCP5 originates from a different organism than the phycobilisomes used in vitro. This point should be clarified.

Indeed, the physiological importance of the majority of HCP homologues in cyanobacteria to photoprotection is still unknown. Here we are not trying to explain these ambiguous results with respect to cyanobacterial physiology; that would be inappropriate, in part because Syn6803 does not encode an HCP, yet Syn6803 PBS can be quenched by HCP5 to an extent beyond that conferred by its cognate OCP. This provides unexpected insight into quenching—to have HCP5 supplement the quenching of OCP1 (at max concentration) indicates there are significant shortfalls in the structural understanding of quenching.

Global expression studies from multiple cyanobacteria suggest that HCPs play a role in stress conditions as summarized in Melnicki *et al*., (2016, Molecular Plant, table 2). In particular, we suggest a photoprotective role of HCP5 based on transcriptomic data in lines 281-284 "*Notably the transcript level of HCP5 in this strain was shown to be up-regulated under medium light which is the theoretical saturating irradiance (1190 μmol*

m-2 s-1) but not under high light (1995 μmol m-2 s-1) when compared with 197 μmol m-2 s-1 (Bernstein et al., 2017)".

The use of HCP5 in combination with OCP1 quenching tricylindrical PBS was to compare their quenching efficiency. The investigation of the physiological impact of HCP5 in an organism that does not contain an HCP homologue like *Synechocystis sp*. PCC 6803 is indeed interesting, however its investigation exceeds the scope of our study.

-The paper novelty seems to lie primarily on the computational investigation rather than the physiological one. The novelty of this study stems from demonstrating that HCP5, unlike other HCP homologs, can induce quenching because of its electrostatics. Therefore, it is essential to substantiate the effectiveness of the quantum mechanics methods used for HCP5. A comparative analysis employing identical methodologies on at least one HCP homolog that does not induce quenching and also on HCP4 which induces quenching, is necessary to convincingly demonstrate that these methods can accurately identify the origins of quenching.

While this manuscript focuses on the quenching capacity of HCP4 and HCP5, we welcome the suggestion as an opportunity to use these data to try to learn something about the enigmatic HCPs 1, 2 and 3 of *Anabaena*.

At the level of primary structure, some important differences are already observable. This is an alignment of the *Anabaena* HCPs, AnOCP1 and Syn6803 OCP1. Residues in close proximity (3 Å) to the PBS in the quenching state of 6803 OCP are shaded in red:

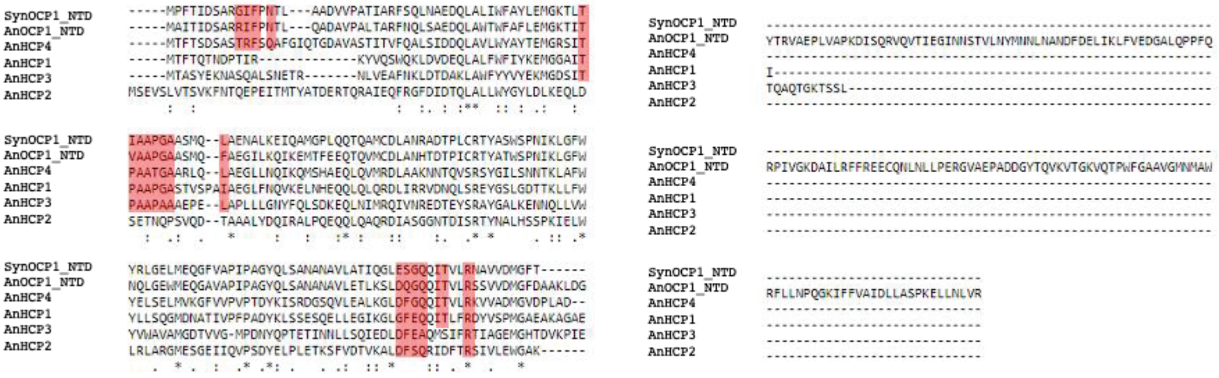

As evident, the N-terminal amino acids important for quenching in SynOCP1_NTD are not found in AnHCP1-3, however are present in AnHCP4. We report the pairwise sequence identity for Anabaena HCP2, HCP4 and the NTD of the AnOCP1 to underscore this point in lines 291-295 "*In contrast, AnHCP2 shows only 38% and 34% sequence identity with AnNTD1 and AnHCP4, respectively. These results add to the enigma surrounding the function of the HCPs; the paralogs typically show distinct regulatory patterns, hinting at distinct functions other than quenching (Giner-Lamia et*

al., 2014; Melnicki et al., 2016; Ho et al., 2017; Yang et al., 2019; Llewellyn et al., 2020; Petrescu et al., 2021; Yang et al., 2023b)".

To explore potential quenching by HCPs further, we generated a homology model of HCP2 bound to the PBS (analogous to those in **Figure 2**) to evaluate its potential quenching with our computational method. This assumes that the HCP2 can bind to the PBS at the known binding site. For other HCPs this seemed implausible; for example, a homology model of HCP1 binding to the PBS is difficult to dock to the PBS due to an additional 2 amino acids (P52+A53, second block of aligned residues) in a region where it interacts closely with the PBS and those two residues cause clashes with the PBS. Accordingly, we pursued the calculations with HCP2, (for which a structure is known showing that the carotenoid is where it would be expected to be); these calculations indicated the TDM is lower (lines 242-248, **Figure S6**), consistent with less likelihood of quenching. These calculations as well as structural clashes at the binding site between HCP2 and the PBS (noted in lines 260-262) are consistent with HCP2's inability to quench.

-The absorption spectra for each sample studied are missing, omitting crucial data for understanding if the spectroscopic properties of either HCP or of the phycobilisomes are altered during complex preparation.

The reviewer makes an important observation. The information provided in **Supplemental Figure S1** shows representative spectra from the purification of HCP4 and HCP5 used in this study.

-The reason for the emission spectra shifting to blue upon quenching induction should be elucidated by the authors, and normalized emission spectra should be provided for clearer understanding.

We appreciate the observation of the reviewer. Indeed, this is a longstanding mystery in the field. Previous reports with native systems (PBS with cognate OCPs/HCPs) have shown that quenched PBS -by either OCP or HCPs- display the blue shift (Wilson, et al., 2012, Plant Cell, Figure 5; Lopez-Igual, et al., 2016, Plant Cell, Figure 4; Bao, et al., 2017, Nature Plants, Figure 6). However, further investigation (probably best begun with a system of PBS and cognate quencher) will need to be undertaken to characterize the basis of this phenomenon which goes beyond the scope of our manuscript at this time.

The emission spectra of PBS are shown using arbitrary units to clearly observe their reduction upon quenching for each experimental condition.

-of major concern, the authors neglect relevant recent literature on OCP, while citing 20 out of 35 references from the works of the corresponding author.

We have added 11 additional references.

30-Aug-2024

Prof. Cheryl A Kerfeld
MSU/LBNL
Biochemistry
East Lansing, Michigan 48824

RE: N-terminal domain homologs of the orange carotenoid protein increase quenching of cyanobacterial phycobilisomes

Dear Prof. Kerfeld:

We are pleased to accept your manuscript for publication in the Plant Physiology. This acceptance is contingent on revision based on the comments of our reviewers. In particular, please consider the point raised by Reviewer #5 about the HCP5-induced quenching.

Please highlight all changes and include a detailed annotation to changes to the text, with line numbers, and noting your responses to the comments.

To submit your revised manuscript, click:

Link Not Available

If you cannot return the revised manuscript within 8 weeks of receipt, please let us know. Otherwise, we will assume that you have elected not to revise the manuscript and are withdrawing it.

Thank you for allowing us to review your work. We look forward to hearing from you soon.

Sincerely,

Editor, Plant Physiology

-- Reviewer comments:
Reviewer #4 (Comments for the Author):

I was not involved in the first round of review of this paper, but I have read the revised submission and the authors' responses to the reviewers' comments. I think the authors have made a good response to the points raised by both reviewers, and the paper provides interesting new information on the roles of the HCPs.

Reviewer #5 (Comments for the Author):

The manuscript of Sheppard et al. reports findings about the ability of N-terminal domain homologs of the OCP to quench cyanobacterial phycobilisome fluorescence. The manuscript is a revision of an earlier submission, which was not evaluated by this reviewer.
Looking at the revised manuscript and the rebuttal of the authors to the previous reviews, the findings look interesting, and the answers are mostly satisfactory (although I have to agree with Reviewer #2 of the earlier version that the quenching ability of the HCPs is not a new discovery).

One of the main statements of the work (Abstract, lines 176-176, 308-309) is that when HCP5 was added after SynOCP1 the quenching of PBS was further enhanced (Fig. 4b), but this effect was not observed when HCP5 was added before SynOCP1 (Fig 4C). Unfortunately, the difference in the fluorescence yields between SynOCP1 and SynOCP1+HCP5 is minimal. A similar difference is shown in Fig. 3 for the sequential addition of HCP4 and AnOCP1, but with the conclusion of no difference. Therefore, the statement about the additional quenching effect (quenching site) of HCP5 is not supported by the data (shown in Fig. 4b) it would need a proper statistical analysis to prove it. Even if the effect would be statistically significant it is so small, that it has only a minor mechanistic relevance (for putative additional binding sites of HCP5).

The authors should provide quantification of the extent of the HCP5-induced additional quenching (from Fig. 4B it looks a few%), with statistical analysis for its significance, or should remove the statement and relevant discussions (which would seriously weaken the significance of the work).

The authors use QM/MM calculations to show that EET couplings between the CAN and the Apc bilins are higher for HCP5 than OCP1 as a support for the higher quenching capacity of HCP5. However, for a general reader of Plant. Phys. it may not be clear how Fig. 7 shows this feature. The maximal density value is higher for both pigments in the case of OCP than HCP5, only the location of the maximum appears at a somewhat higher coupling value for HCP5. These features should be explained more clearly.

--- Reviewer comments:
Reviewer #4 (Comments for the Author):

I was not involved in the first round of review of this paper, but I have read the revised submission and the authors' responses to the reviewers' comments. I think the authors have made a good response to the points raised by both reviewers, and the paper provides interesting new information on the roles of the HCPs.

Reviewer #5 (Comments for the Author):

The manuscript of Sheppard et al. reports findings about the ability of N-terminal domain homologs of the OCP to quench cyanobacterial phycobilisome fluorescence. The manuscript is a revision of an earlier submission, which was not evaluated by this reviewer.
Looking at the revised manuscript and the rebuttal of the authors to the previous reviews, the findings look interesting, and the answers are mostly satisfactory (although I have to agree with Reviewer #2 of the earlier version that the quenching ability of the HCPs is not a new discovery).

One of the main statements of the work (Abstract, lines 176-176, 308-309) is that when HCP5 was added after SynOCP1 the quenching of PBS was further enhanced (Fig. 4b), but this effect was not observed when HCP5 was added before SynOCP1 (Fig 4C). Unfortunately, the difference in the fluorescence yields between SynOCP1 and SynOCP1+HCP5 is minimal. A similar difference is shown in Fig. 3 for the sequential addition of HCP4 and AnOCP1, but with the conclusion of no difference. Therefore, the statement about the additional quenching effect (quenching site) of HCP5 is not supported by the data (shown in Fig. 4b) it would need a proper statistical analysis to prove it. Even if the effect would be statistically significant it is so small, that it has only a minor mechanistic relevance (for putative additional binding sites of HCP5).

We appreciate the reviewer's request to clarify our findings. The main objective of the **Figure 4** panels B and C is to demonstrate that the maximum capacity of HCP5 to quench the PBS cannot be surpassed by the addition of photoactivated *Syn*OCP1, whereas the maximum PBS quenching yielded by photoactivated *Syn*OCP1 is actually surpassed by the addition of HCP5. This implies that either (i) HCP5 can quench PBS at sites where SynOCP1 cannot bind, (ii) HCP5 quenching is higher than *Syn*OCP1 or (iii) both (i) and (ii). We indicate our reasoning in lines 173-177: "*To determine if SynOCP1^R and HCP5 bind to the same site on the tricylindrical PBS, we assayed them in combinations. Surprisingly, when SynOCP1^R was used to quench SynPBS with subsequent addition of HCP5, quenching was further enhanced (**Figure 4B**). However, in the converse experiment in which HCP5 quenching was followed with the addition of SynOCP1^R, no additional quenching was observed (**Figure 4C**)*".

The authors should provide quantification of the extent of the HCP5-induced additional quenching (from Fig. 4B it looks a few %), with statistical analysis for its significance, or

should remove the statement and relevant discussions (which would seriously weaken the significance of the work).

We agree with the reviewer's comment that this is an excellent augmentation of our description of this surprising result and have added an extra panel in **Figure 4** for the quantification of the PBS quenching capacity yielded by using a maximum concentration of HCP5 or *Syn*OCP1. We can confidently say that the difference between HCP5 and *Syn*OCP1 is statistically significant (using three biological replicates, with a P value below 0.005). When using the same analysis for the comparison of HCP4 and *An*OCP1 we note that this difference is not statistically significant (also with three biological replicates giving a P value above 0.1). We further clarify the difference between HCP5 and *Syn*OCP1 in lines 178-179: "*Comparison of the maximum PBS quenching percentage shows that indeed HCP5 displays higher quenching capacity compared to SynOCP1^R (**Figure 4D**)".*

The authors use QM/MM calculations to show that EET couplings between the CAN and the Apc bilins are higher for HCP5 than OCP1 as a support for the higher quenching capacity of HCP5. However, for a general reader of Plant. Phys. it may not be clear how Fig. 7 shows this feature. The maximal density value is higher for both pigments in the case of OCP than HCP5, only the location of the maximum appears at a somewhat higher coupling value for HCP5. These features should be explained more clearly.

We thank the reviewer for their suggestion. In **Figure 7** we show a distribution of EET couplings, which is shifted towards larger coupling values in HCP5. In turn, this means that the average couplings are larger in HCP, meaning that EET quenching will be faster. We changed the last paragraph of the Results section to better explain that **Figure 7 B, C** shows a distribution of coupling values, and we now include the change in the average coupling in lines 267-272 "***Figure 7B** and **C** show the distribution of the couplings with both pigments, which clearly peaks at larger coupling values (**Figure 7B and C**). The higher proportion of HCP5 structures with large couplings reflects the increased TDM of the dark state. The average coupling increases by about 25% for $ApcA_1$ (56 vs 45 cm^{-1}) and $ApcA_2$ (34 vs 27 cm^{-1}), implying a faster EET in HCP5-PBS, and thus a more efficient quenching*".

17-Sep-2024
Prof. Cheryl A Kerfeld
MSU/LBNL
Biochemistry
East Lansing, Michigan 48824

MSID: PP2024-RA-01066DR1
MS TITLE: N-terminal domain homologs of the orange carotenoid protein increase quenching of cyanobacterial phycobilisomes

Dear Prof. Kerfeld:

I am pleased to inform you that your revised manuscript "N-terminal domain homologs of the orange carotenoid protein increase quenching of cyanobacterial phycobilisomes" has been accepted for publication in Plant Physiology. Your manuscript will be checked for consistency with journal formatting and image standards by one of our Science Editors, who will contact you shortly. They may have a number of queries and suggestions for you. Please keep in mind that they work to increase the online visibility of your article and its attraction for readers from the widest of backgrounds. You will have the opportunity to review any revisions, and I hope you will work constructively with them to get the most out of your research. You will also have the opportunity to review all changes when you receive the author proofs for your article.

Once validated for publication, your article will be sent to a Science Editor for a final review of your manuscript and source files. After your final files are uploaded, your article will appear in the next available issue and the online Preview version of your article will be posted after your signed Copyright and Checklist forms and final source files are received.

Please be sure to include the following in your manuscript file:
Harvard style citations
List of all author contributions and funding information at the beginning of manuscript after the titles and author list.
List all supplemental materials associated with the article, with titles, at the end of the ms, before the references and acknowledgments section.
Complete Figure Legends for all figures appearing in the article listed before the Literature Cited.

LICENSE INFORMATION Shortly after your paper is sent to production, you will receive an email from our publisher's author support team, **SciPris**, with information on article processing charges, as well as the types of Open Access license you would be able to purchase. The accepted version of your manuscript will not appear on our Advance Articles page until the license is selected and signed, so please look out for an email from SciPris. For more information see the Article Publication Fees section of our author guidelines: https://academic.oup.com/plcell/pages/General-Instructions

Finally, we encourage your submission of artwork for the journal cover. If there is an image or illustration related to your paper that you would like to have considered, please email it in .pdf or .tif format to clowe@aspb.org. An image title, brief, 2-3 sentence description of the image, and the name of the person credited for the image are also encouraged. If your image is selected to appear on the online cover of the journal, you will be contacted with the scheduled issue date and further information.

Thank you and congratulations on your Plant Physiology paper!

Sincerely,
Editor, Plant Physiology

=====
FIRST AUTHOR PROFILES
=====

We recognize the hard work that goes into being First Author of a Plant Physiology paper by publishing a "First Author Feature" for each First Author alongside your paper.

If the first author(s) would like to submit information for a feature on Facebook and Twitter, fill out the form by clicking on the link below:

Plant Physiology First Author Feature Form: https://forms.office.com/r/ZBCGZfhm8F
If you cannot access the form through the link above, please contact Rachel at rbelsky@aspb.org.

To upload an image of yourself to accompany your profile, please put your photo in the correct journal folder at the link below. You must label your image: Author First Name Last Name Article Title.

First Author Profile Image Dropbox: https://www.dropbox.com/scl/fo/xz029p7qjr2jml2rp2kmq/h?dl=0&rlkey=6fkxcaqmxx35ap152b2osy1fz

=====
IMPORTANT REMINDER: PEER REVIEW REPORTS
=====

If you opted to publish a peer review report along with your article during the original submission process, it will be prepared by the editorial staff and publicly posted with your manuscript, inside the zip file that contains any other supplemental material. As a reminder, the peer review report is a public record of all comments from editors and reviewers, as well as your prior responses, as you received them in the decision letters for each draft of your manuscript. If you agreed to publish this report and have changed your mind, or are not sure if you selected this option, please contact the editorial office as soon as possible before signing the license agreement from our publisher.

-- Reviewer comments:
